# Insights into the circulating microbiome of Atlantic and Greenland halibut populations: the role of species-specific and environmental factors

**DOI:** 10.1038/s41598-023-32690-6

**Published:** 2023-04-12

**Authors:** Fanny Fronton, Sophia Ferchiou, France Caza, Richard Villemur, Dominique Robert, Yves St-Pierre

**Affiliations:** 1 INRS-Centre Armand-Frappier Santé Technologie, 531 Boul. des Prairies, Laval, QC H7V 1B7 Canada; 2 Institut des Sciences de la Mer, Université du Québec à Rimouski, 310, allée des Ursulines, C.P. 3300, Rimouski, QC G5L 3A1 Canada

**Keywords:** Ecology, Ecology

## Abstract

Establishing long-term microbiome-based monitoring programs is critical for managing and conserving wild fish populations in response to climate change. In most cases, these studies have been conducted on gut and, to a lesser extent, skin (mucus) microbiomes. Here, we exploited the concept of liquid biopsy to study the circulating bacterial microbiome of two Northern halibut species of economic and ecological importance. Amplification and sequencing of the 16S rRNA gene were achieved using a single drop of blood fixed on FTA cards to identify the core blood microbiome of Atlantic and Greenland halibut populations inhabiting the Gulf of St. Lawrence, Canada. We provide evidence that the circulating microbiome DNA (cmDNA) is driven by genetic and environmental factors. More specifically, we found that the circulating microbiome signatures are species-specific and vary according to sex, size, temperature, condition factor, and geographical localization. Overall, our study provides a novel approach for detecting dysbiosis signatures and the risk of disease in wild fish populations for fisheries management, most notably in the context of climate change.

## Introduction

The Atlantic halibut *Hippoglossus hippoglossus* (Linnaeus, 1758) *(H. hippoglossus)* and the Greenland halibut *Reinhardtius hippoglossoides* (Walbaum, 1792) *(R. hippoglossoides)* are two species of flatfish widely distributed in the Northwest Atlantic and characterized by distinct populations in the Gulf of St. Lawrence (GSL)^1,2^. These populations support the region's two most valuable groundfish fisheries and are biannually assessed to provide scientific advice for management^3,4^. The Atlantic halibut abundance has been steadily increasing, whereas the Greenland halibut stock has declined over the last two decades. Although the reasons for these fluctuations are not fully clear, the rapid warming of the deep channels of the GSL^3,5^ and increased competition by redfish for Greenland halibut^6,7^ are considered the primary factors driving these changes. Given the impact of such changes on the physiology of the fish, developing sensitive and predictive biomarkers is essential for a close follow-up of the health status of these stocks.

The microbiome, or the pool of nucleic acids from microbes found in/on a host species, has attracted considerable attention from scientists in recent years as a predictive biomarker to assess the health status of an individual^8^. The microbiome is an important determinant of the health status of an organism, as it contributes to the regulation of several physiological processes, such as the immune response and host energy metabolism.

Although most studies on the use of microbiome signatures as a predictive tool were initially performed in clinical settings, the increased accessibility of next-generation sequencing (NGS) technologies for the analysis of 16S ribosomal RNA (rRNA) gene amplicons has facilitated its application in different research fields, including studies in fish populations^9^. Studies on fish's skin and gut bacterial microbiomes have shown that a balanced microbiome plays a critical role in the host’s health, protecting against pathogens while bringing nutritional benefits^10^. Disruption of this balance, often called dysbiosis, changes the biodiversity and abundance of specific bacterial communities, often leading to health complications^11^.

Considering their economic and ecological importance, an increasing number of studies have thus focused on defining the microbiome signatures of Teleost. These studies have shown that microbiome signature depends on several factors, including host genetics, morphometrics, and several environmental factors, including biotic and abiotic factors^12,13^. In most cases, however, these studies have been conducted in laboratory/experimental settings or fish farms and performed on the gut microbiome and, to a lesser extent, skin (mucus) microbiomes^13–15^. In recent years, however, the concept of a circulating microbiome has emerged as an interesting alternative to invasive, lethal, and logistically challenging tissue biopsies. Even if blood has historically been considered exempt from microbes in healthy individuals, it is now irrefutable that bacterial, viral, fungal and other microorganism genomes are present in the blood (blood-cell or plasma)^16–20^. This feature allows us to study the microbiota of an organism without the need for tissue biopsy. The concept of the circulating microbiome is particularly well adapted to the development of routine and predictive biomarkers. The utility of this approach has recently been demonstrated in clinical settings, offering a new perspective for the development of biomarkers in ecology^21–24^ similar to those described for several diseases in humans^16,20,25^. In fact, the existence of a blood microbiome is a concept that is now widely accepted in humans and animals, including pigs, broiler chickens, camels, cows, goats, cats and dogs^21,23,24,26–29^.

In the present work, we have characterized, for the first time, the blood 16S rRNA microbiome signatures of two wild fish populations of ecological and economic interest from the GSL, the Atlantic halibut and the Greenland halibut*.* These two species are characterized by opposite abundance trends*.* Our general hypothesis is that physiological and environmental factors impact their microbiome signature. By providing a reference for future studies to examine climate change's impact on halibut populations, we further hypothesize that our approach will help develop novel biomarkers for monitoring the condition and health of wild fish populations.

## Material and methods

### Sampling

**Figure 1:**
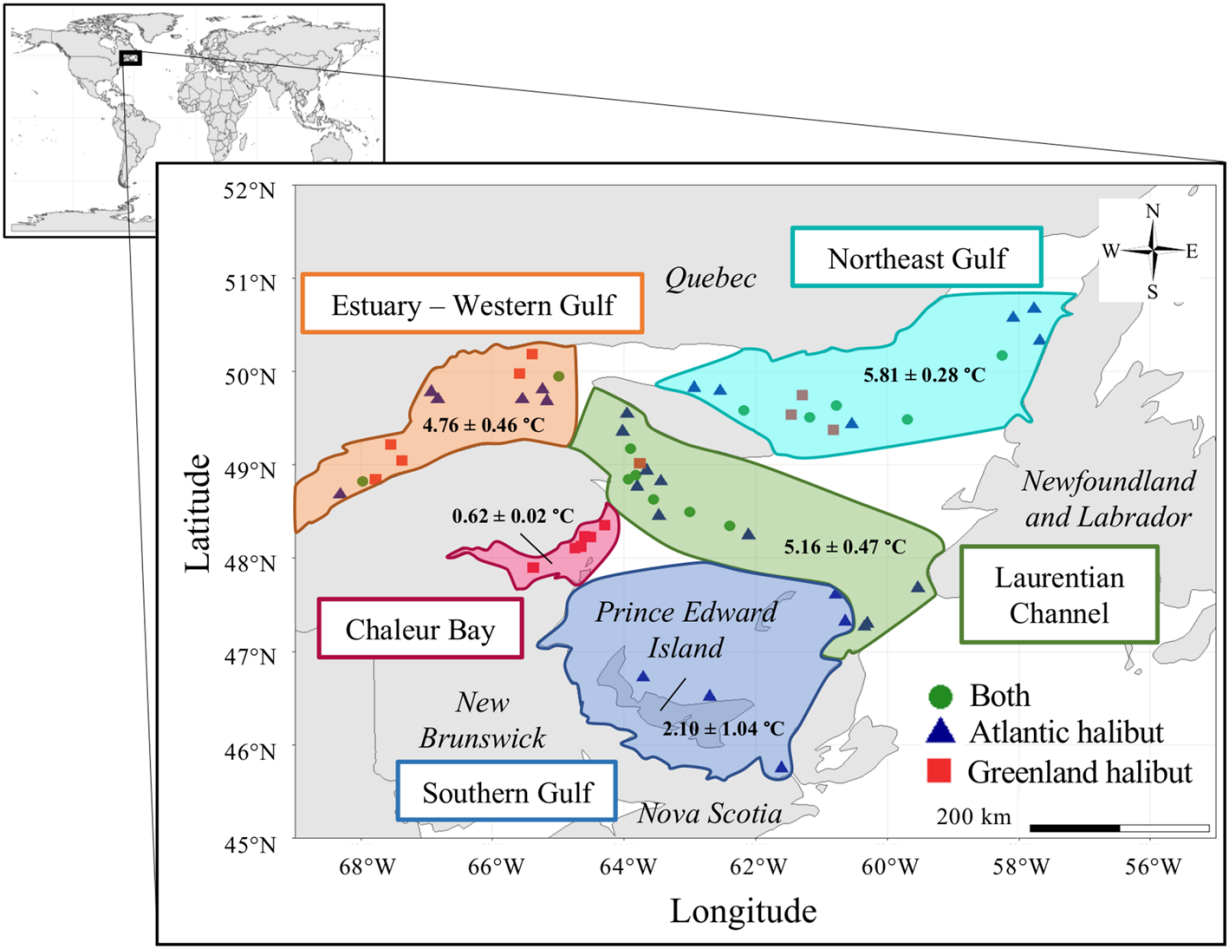
Map of the sample sites divided into five different areas. The regional temperature is given for each zone (mean ± SE).

Blood samples from Greenland halibut (n = 97; length: 316.2 ± 15.1 mm) and Atlantic halibut (n = 86; 762.0 ± 30.1 mm) were collected between August 15th and October 1st, 2019, during the annual bottom trawl surveys performed on the northern and southern sectors of the GSL (Canada) by the Department of Fisheries and Oceans (DFO) (Table 1). Scanmar hydroacoustic sensors attached to the trawl and a conductivity, temperature, and depth (CTD) probe were used to record the temperature. Blood samples were taken immediately upon trawl retrieval, and liquid biopsies were performed at 56 sites for at least one species (Fig. 1). The number of liquid biopsies performed per station (ranging from 1 to 14) was opportunistic and depended on the presence of either halibut species and workload at a given site. Blood samples were collected with a heparin-coated 3-mL sterile syringe and a 22-G needle following a dorsal incision using a sterilized knife. Drops of blood were collected and immediately stored on a Flinders Technology Associates (FTA) card (Sigma-Aldrich, Oakville, ON, Canada). Samples were allowed to air dry and kept in a plastic bag with a desiccant, as Caza et al. (2019) described. The sex of each sampled individual was determined by visual identification of the gonads following the dissection of specimens by the DFO science crew. The care and use of field-sampled animals complied with the Government of Canada's animal welfare laws, guidelines, and policies approved by Fisheries and Oceans Canada. All methods were conducted following ARRIVE guidelines (https://arriveguidelines.org/).

**Table 1:** Summary of the fish samples used for cmDNA analysis.

Species	Sex	N	Length (mm) mean ± SE	Weight (g) mean ± SE
Greenland halibut	Total	97	316.2 ± 15.1	518.4 ± 72.2^a^
	Male	19	341.2 ± 23.7	407.1 ± 67.5
	Female	32	433.0 ± 22.7	915.1 ± 125.8
	Unknown	46	224.5 ± 17.0	37.6 ± 1.6^b^
Atlantic halibut	Total	86	762.0 ± 30.1^c^	6 976.8 ± 879.7^c^
	Male	55	755.1 ± 36.4^d^	6 628.5 ± 871.0^d^
	Female	30	760.3 ± 54.6	7 167.8 ± 1935.6
	Unknown	1	1 190	20 060

Number of samples for which the data were available: ^a^n = 73, ^b^n = 22, ^c^n = 85 and ^d^n = 54.

### DNA extraction, amplification and sequencing

All DNA extraction and purification procedures were conducted in a white room where pressure, temperature, and humidity were controlled to minimize contamination. Individual discs were cut from the FTA cards using a sterile 5.0-mm single round hole punch, and total DNA was isolated using the QIAamp DNA Investigator Kit (Qiagen, Toronto, ON, Canada), according to the manufacturer’s protocol. DNA was quantified in duplicate using a Quant-iT PicoGreen dsDNA detection kit (Molecular Probes, Eugene OR, USA). Amplification of the V3–V4 region of the 16S ribosomal RNA (rRNA) gene and 16S gene amplicon sequencing for all DNA samples were performed at Centre d'Expertise et de Services, Génome Québec (Montréal, QC, Canada). Amplification used the universal primers 341F (5′-CCTACGGGNGGCWGCAG-3′) and 805R (5′-GACTACHVGGGTATCTAATCC-3′). Sequence libraries were prepared by Genome Quebec with the TruSeq DNA Library Prep Kit (Illumina, San Diego, CA, USA) and quantified using the Kapa Library Quantification Kit for Illumina platforms (Kapa Biosystems). Paired-end sequences were generated on a MiSeq platform PE300 (Illumina Corporation, San Diego, CA, USA) with the MiSeq Reagent Kit v3 using 600 cycles (Illumina, San Diego, CA, USA). Raw data files are publicly available on the NCBI Sequence Read Archive (PRJNA853332).

### 16S rRNA data processing

Illumina sequence data (FASTQ files) were trimmed using *Cutadapt* (version 2.8). The 16S rRNA (V3-V4) amplicon sequence variants (ASVs) were generated with the DADA2 pipeline (version 1.16.0; Callahan et al*.* (2016)) and subsequently within the R environment (R version 4.0.3, Team (2021)). The Ribosomal Database Project (RDP)16 database was used for the ASV assignment. The software packages *phyloseq* (1.36.0)^30^, *microbiomeSeq* (0.1)^31^, *microbiomeMarker* (0.99.0)^32^, and *vegan* (2.5.7)^33^ were used to characterize the microbial communities. The maps were created with the packages *ggplot2* (3.3.6)^34^ and *rnaturalearth* (0.1.0)^35^. An ASV was considered part of the core microbiome if it had a minimum prevalence (rate of presence in the group of samples) of 70% with a detection threshold of 0.01% relative abundance^36^. A similar decision tree was applied for the core genus (abundance of ASVs of the same genus were summed) but with 90% prevalence. A stringent threshold of 90% was chosen, as reported in previous studies^37–39^. The prevalence varies greatly between microbiome studies, ranging from > 10 to 100% ^22,36,37,39–42^. Hence, we chose not to limit the core microbiome at 100% prevalence because the study was not under controlled conditions; instead, it was performed in wild populations where greater variation was expected, especially with sample sizes considered.

### Classes based on environmental and morphometric data

Individual fish were classified to assess environmental and ontogenetic effects. They were assigned as mature or immature according to the known length at which 50% of males had reached reproductive size (L50), i.e., 360 mm for Greenland halibut^3,7^ and 920 mm for Atlantic halibut^43^. Given that sex information was missing from many individuals, the male L50 was chosen, as it is lower, so the mature individuals would have less chance to be mislabeled as immature. Because diet composition is a likely factor influencing the microbiome, we split Greenland halibut specimens into four size classes based on body lengths where major shifts in diet composition have previously been described^44^. Briefly, four classes were defined for the Greenland halibut: (1) Class 1, individuals smaller than 200 mm feeding on small prey; (2) Class 2, individuals ranging from 200 to 400 mm feeding on intermediate prey; and (3) Class 3, individuals larger than 400 mm feeding on large prey. Defining length classes for the Atlantic halibut was impossible, as the number of small and large individuals was too low. Classes were also defined according to the water temperature. Individuals occupying temperatures below than 5 °C were considered “cold water”, and those occupying temperatures above 5 °C were considered “warm water”^7^. Given that Atlantic halibuts’ range of temperature tolerance is wider than those measured, they were not included in temperature-based analyses^45,46^. Finally, the relative condition K factor, a broad health index for fish, was also calculated based on the length and weight of each individual^47,48^. A linear regression was performed between log_10_ (weight) and log_10_ (length) as follows:

$\log_{10} \left( W \right) = \log_{10} \left( a \right) + b*\log_{10} \left( L \right)$^49^.where W is the weight, L is the length, and a and b are constant coefficients.

The coefficients a and b were calculated and used to estimate the expected weight W_e_ of each individual based on their length with the following equation:

$$W_{e} = aL^{b}$$

Finally, the K_rel_ of Le Cren was calculated as follows:

$$K_{rel} = \frac{W}{{W_{e} }}$$

The comparison between the individual's actual weight and their expected weight gives us the fish's plumpness, a glimpse of its health status. Basically, if an individual is skinnier than others of the same length within the same species, it is under stress, and K_rel_ will be under 1. On the contrary, a plump fish is associated with beneficial environmental influence, and K_rel_ will be over 1^50^.

The autocorrelation, the homoscedasticity of the error terms and the normal distribution of the linear regression residuals were validated with statistical analysis (Durbin Watson, Breusch‒Pagan and Shapiro‒Wilk tests). Classes were created with K_rel_ > 1 corresponding to the “high condition” and K_rel_ < 1 corresponding to the “low condition”^47,50^.

### Spatial analysis

Overall, five geographical zones were defined. Spatial zones were created to separate different habitats in the Gulf based on sample sites with similar characteristics as follows (e.g., depth, temperature, and spatial closeness); The Estuary-Western Gulf area extends from 65°W to 69°W longitude and from 49.7°N to 51°N latitude, considered to be the primary nursery area for the Greenland halibut^51^, the Northeast Gulf area, situated between 57°W and 63.7°W longitude and 49°N and 51°N latitude; the Lawrence Channel area is between longitudes 59°W and 65°W and latitudes 47°N and 49.7°N with sample sites deeper than 110 m (included); the Chaleur Bay area includes sampling sites between 64°W and 67°W longitude and 47.5°N and 48.5°N latitude, and finally, the Southern Gulf area is located between 60.5°W and 65.5°W longitude and 45.5°N and 48°N latitude with sampling sites less deep than 110 m (not included). For Greenland halibut, the number of fish per area was n = 22 for the Estuary-Western Gulf t, n = 19 for the Chaleur Bay, n = 22 for the Northeast Gulf and n = 34 in the Laurentian Channel. Regarding Atlantic halibut, the number of fish was n = 14 for the Estuary-Western Gulf, n = 11 for the Southern Gulf, n = 17 for the Northeast Gulf and n = 44 for the Laurentian Channel.

### Statistical analysis

Bacterial taxonomic α-diversity (intrasample) was estimated using the richness and the Shannon and Simpson indices, as implemented in the R package *microbiome* (1.14.0)^52^. Variations in bacterial α-diversity and taxa abundances between the two species were assessed using either the Kruskal‒Wallis test or Wilcoxon-Mann‒Whitney test, given that none of the variables exhibited a normal distribution. In addition, α-diversity was also calculated among classes according to the length, temperature and condition factor K^48^. The Kruskal‒Wallis test was followed by a pairwise Wilcoxon-Mann‒Whitney test if the *p*-value (*p*) was significant (*p* < 0.05). The β-diversity (intersample) was estimated using phylogenetic weighted UNIFRAC dissimilarities assessed by principal coordinates analysis (PCoA). Differences in community composition were tested using permutational multivariate analysis of variance (PERMANOVA) for weighted UNIFRAC indices with 999 permutations, as implemented in the R *vegan* package (2.5.7) or the *pairwise Adonis* package (0.4). Detailed statistical analyses on variations with morphometric and environmental data are presented in Supplementary Information. Differences were considered statistically significant at alpha = 0.05. Linear discriminant analysis (LDA) effect size (LEfSe) was performed on the microbiome of each species and on the different classes to highlight discriminative taxa for each class. This analysis was performed using the *microbiomeMarker* package (0.99.0). The cutoff was chosen at an LDA score of log_10_ (LDA score) ≥ 4. All analyses were performed in R studio (v4.0.5)^53^.

## Results

### Preliminary characterization of the cmDNA

A total of 183 Atlantic and Greenland halibut blood samples were collected at the end of summer and early fall of 2019 in the Gulf of St. Lawrence (Fig. 1). The cmDNA signatures were determined by sequencing the V3-V4 hypervariable regions of the 16S rRNA gene. Approximately 6 million raw reads were retrieved after filtering (2.5 and 3.5 million for Atlantic and Greenland halibut, respectively). The number of sequences per sample ranged between 3,985 and 55, 077. The mean numbers of reads per individual were 35,575 ± 971 and 29,296 ± 1 185 for Greenland halibut and Atlantic halibut, respectively. The number of ASVs per sample curve confirmed that the sequencing depth was sufficient to plateau the number of ASVs (Figure S1). A total of 7,105 unique ASVs were obtained, including 7,102 identified as having bacterial origins (3 of archaeal origin were removed from the analysis). A total of 6,450 ASVs were greater than or equal to 0.01% in relative abundance. Overall, 3,362 ASVs were present in Atlantic halibut (mean = 112 ± 7 per individual) and 5,023 in Greenland halibut (mean = 161 ± 9 per individual).

### Differences in the circulating microbiome at the phylum level

**Figure 2:**
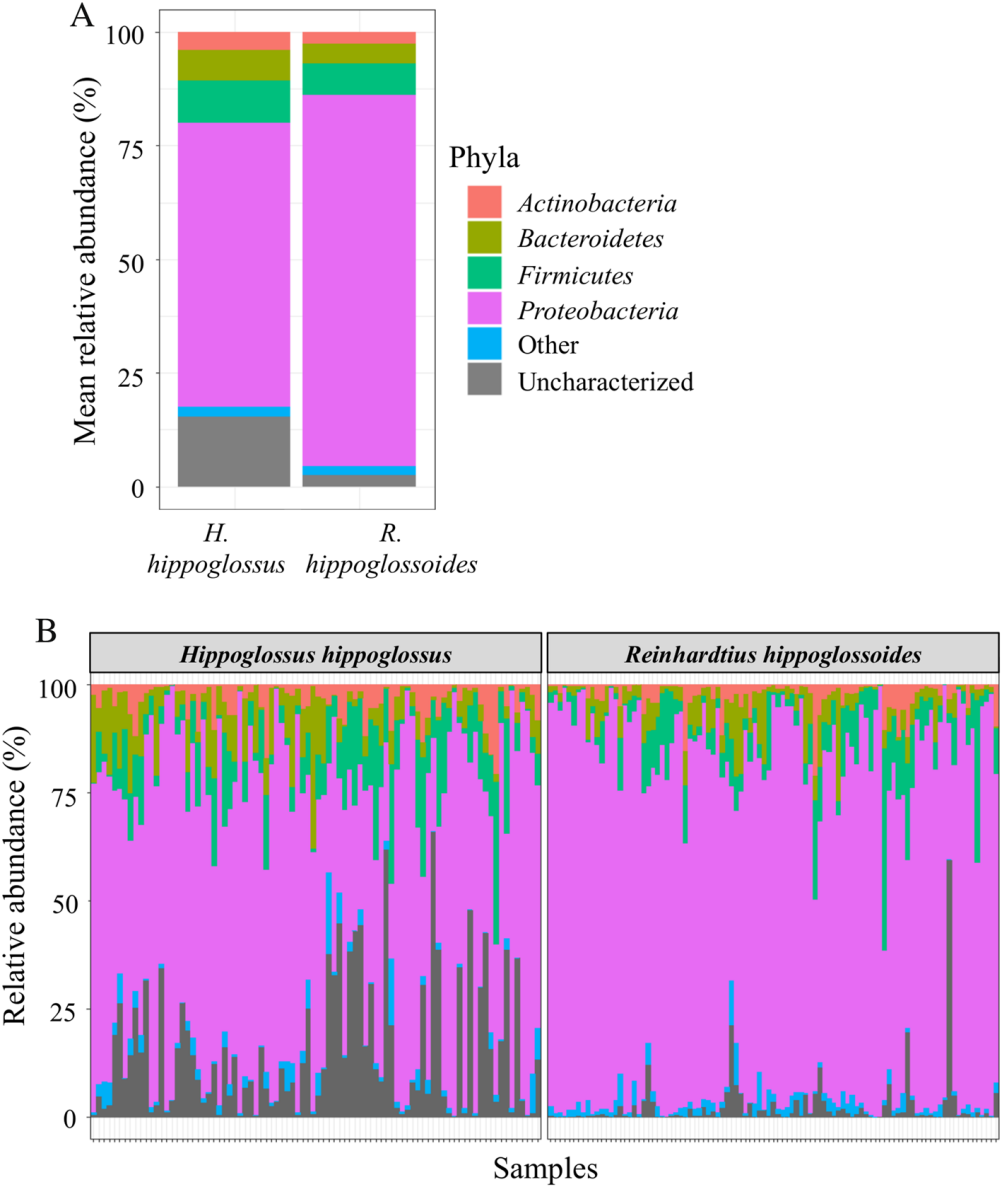
Microbiome structure at the phylum level. (**A**) Mean relative abundance (%) of the four main phyla present in the blood microbiome of Atlantic halibut (*H. hippoglossus*) and Greenland halibut (*R. hippoglossoides*). (**B**) Individual variation in the relative abundance (%) of the main phyla of Atlantic halibut (n = 86) and Greenland halibut (n = 97).

Given both halibut species' genetic and behavioral differences, we first tested the hypothesis that the microbiome differs between the Atlantic and Greenland halibut. Overall, 30 different phyla were identified (23 and 29 in Atlantic halibut and Greenland halibut, respectively), 63 classes (44 and 59), 121 orders (88 and 112), 241 families (189 and 224) and 685 genera (473 and 587) (Table 2). At the phylum level, the blood microbiome signature was dominated by *Proteobacteria*, *Firmicutes*, *Bacteroidetes*, and *Actinobacteria* (Fig. 2). The mean relative abundance of *Proteobacteria* was, however, significantly higher in Greenland halibut (81.6 ± 1.5% versus 62.4 ± 2.3% in Atlantic halibut) (Wilcoxon-Mann‒Whitney test; *p* < 0.001). Similarly, the other three main phyla were significantly higher in Atlantic halibut at 9.3 ± 0.9% versus 6.9 ± 0.8% for *Firmicutes* (*p* = 0.032), 6.6 ± 0.7% versus 4.4 ± 0.5% for *Bacteroidetes* (*p* = 0.044) and 4.0% ± 0.5% versus 2.6 ± 0.4% for *Actinobacteria* (*p* = 0.0028) (Fig. 2A). The mean relative abundance of uncharacterized bacteria was nonetheless important in Atlantic halibut, accounting for ~ 15% on average. This finding is even more apparent based on the individual relative abundance, with some individuals possessing 60% of uncharacterized bacteria (Fig. 2B). The microbiome structures with other taxa are provided in Supplementary Figures S2-S3.

**Table 2:** Number of taxa in the cmDNA of the Atlantic halibut (*H. hippoglossus*) and the Greenland halibut (*R. hippoglossoides*).

Taxon	Atlantic halibut (n = 86)	Greenland halibut (n = 97)	Total
Phylum	23	29	30
Class	44	59	63
Order	88	112	121
Family	189	224	241
Genus	473	587	685

### The genus-level core circulating microbiome

**Figure 3:**
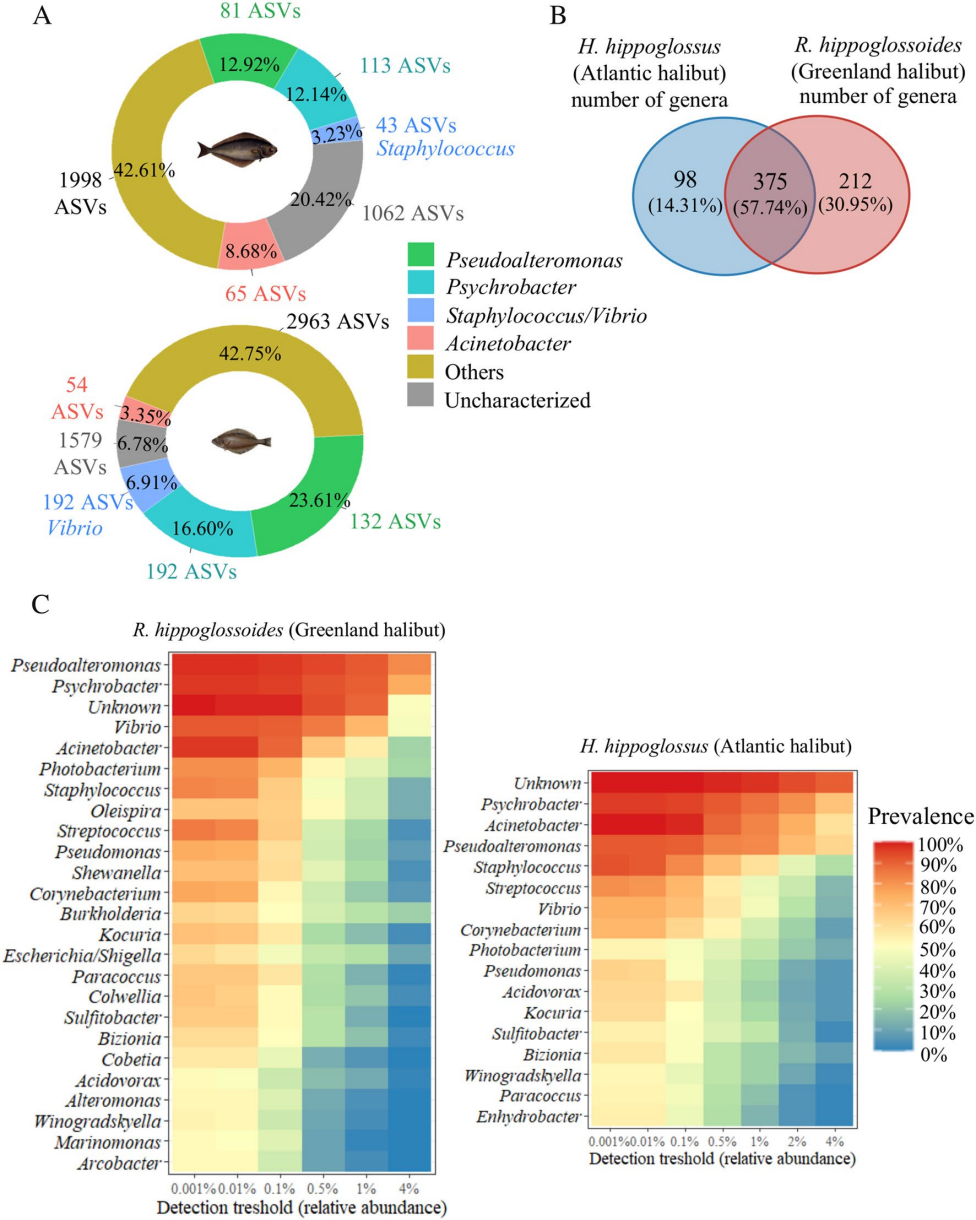
Core blood microbiome analysis. (**A**) Mean relative abundance (%) of the core genera aggregated (90% prevalence) in the cmDNA of the blood microbiome of the Atlantic halibut (*H. hippoglossus*) (top) and the Greenland halibut (*R. hippoglossoides*) (bottom). The mean relative abundance is given in each pie chart, and the number of aggregated ASVs is indicated next to each pie chart. (**B**) Venn diagram showing common and distinctive genera in the blood microbiome. (**C**) Heatmaps of the core microbiome. These heatmaps identify the most prevalent bacteria in both halibut species. Atlantic halibut, n = 86, Greenland halibut, n = 97.

To further test the hypothesis that the microbiome signature of both species differs, we carried out a detailed analysis at the genus level. Three genera, *Pseudoalteromonas, Psychrobacter* and *Acinetobacter,* were present in 90% of samples for both species (ASVs were aggregated together at the genus level). In Atlantic halibut, these genera were found to be the most abundant, accounting for 12.9%, 12.1% and 8.7% of the mean relative abundance, respectively. A fourth genus that was present in 90% of samples was *Staphylococcus,* with a mean relative abundance of 3.2% (Fig. 3A). In Greenland halibut, these three genera represented 23.6%, 16.6% and 3.4% of the average blood microbiome, respectively, whereas *Vibrio* was the fourth core genus with 6.9% mean relative abundance. In total, 685 genera were found, 375 of which were present in both halibut species (54.7%) (Fig. 3B). Although most of the genera found at 50% prevalence were shared by both species, some genera were unique in each species, such as *Enhydrobacter* in Atlantic halibut and *Oleispira* in Greenland halibut (Fig. 3C). This distribution of genera differed between the two species; major differences were the higher abundance of *Vibrio* and the lower abundance of *Acinetobacter* in Greenland halibut. The number of genera with a prevalence of 50% was also higher in Greenland halibut than in Atlantic halibut.

### Comparative analysis of the bacterial diversity between species

**Figure 4:**
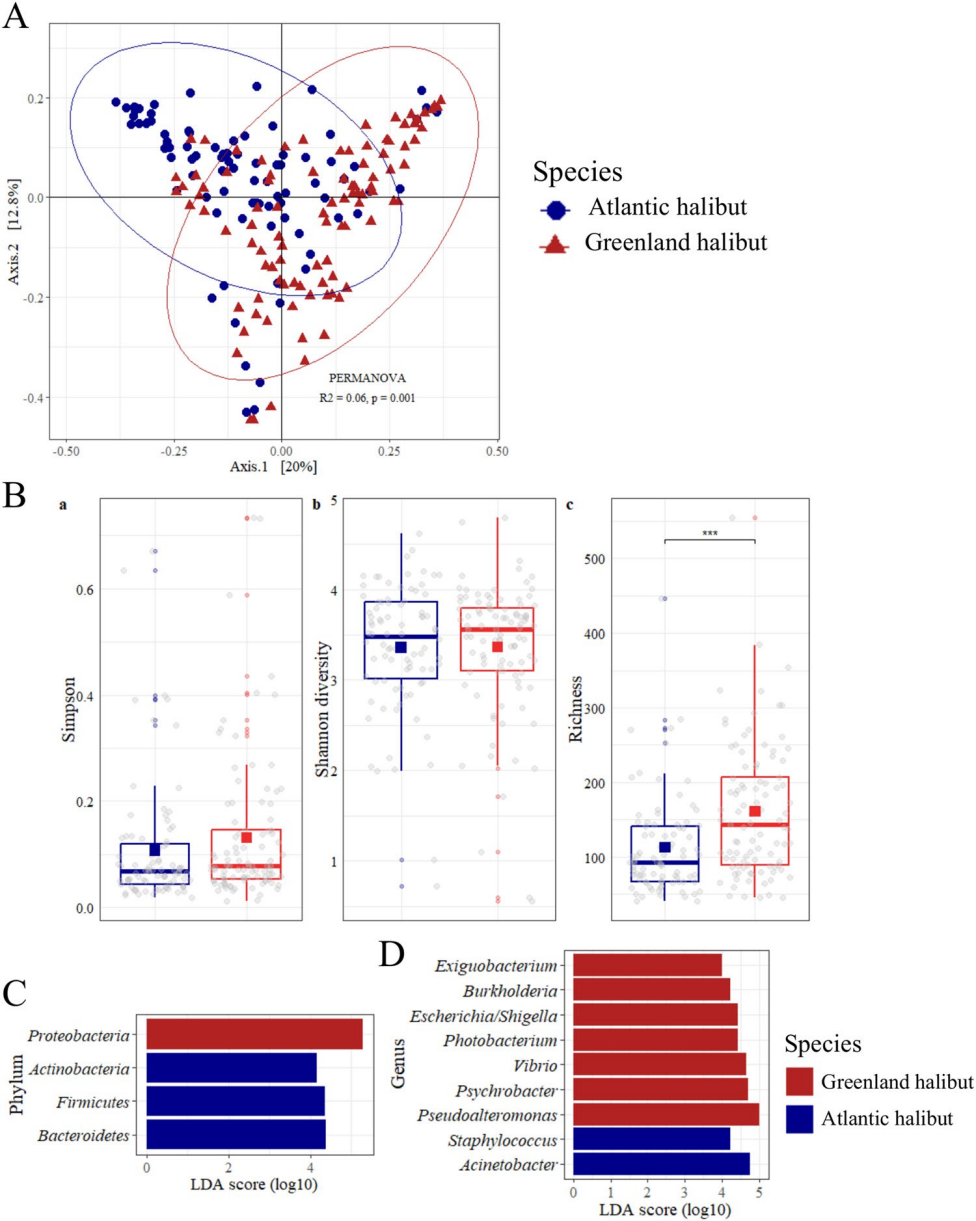
Biodiversity analysis. (**A**) PCoA plot of the β-diversity of the blood microbiome based on weighted UniFrac distances. (**B**) α-Diversity metrics for the cmDNA of Atlantic halibut (blue) and Greenland halibut (red). ***: *p* < 0.001. (**C** and **D**). Discriminative taxa at the phylum and genus levels in the cmDNA of both halibut populations (*p* < 0.05). Atlantic halibut, n = 86, Greenland halibut, n = 97.

We next compared the overall biodiversity of the cmDNA between both species using a UniFrac-based PCoA that showed that the microbiomes tended to cluster according to species, but with high variations within species composing the microbiome and a low percentage of variance explained by the two axes (32.8%) (Fig. 4A). PERMANOVA confirmed the significant difference between the two halibut species (*p* < 0.001), but the effect size was very low for the groups (R^2^ = 0.06) compared to the residuals (R^2^ = 0.94), suggesting that the species of the individual was not a strong determinant between these fish microbiomes. These findings support a recent study showing that the gut microbiome of flounders (*Pleuronectidae)* is similar among family members (Huang et al., 2020). Regarding α-diversity indicators, no significant differences in evenness or diversity were noted between the two species; the mean Simpson index (Atlantic halibut, 0.11; Greenland halibut*,* 0.13) and the mean Shannon diversity index (Atlantic halibut 3.36; Greenland halibut, 3.37) were equivalent (Fig. 4B). The richness index, however, was higher in the case of Greenland halibut, with an average of 161 ± 9 ASVs per individual compared to 112 ± 7 ASVs per individual for Atlantic halibut (*p* < 0.001).

### LEfSe comparison of the two species

To further investigate the differences between the circulating microbiomes of the two halibut species, a Linear discriminant analysis Effect Size (LEfSe) was performed with a cutoff at log_10_ (LDA score) ≥ 4. This criterion allowed us to observe several differences in the structure of the two blood microbiomes at the phylum and genus levels (Fig. 4C and D). In particular, *Proteobacteria* were found in higher abundance in Greenland halibut, whereas *Actinobacteria*, *Firmicutes* and *Bacteroidetes* were more abundant in Atlantic halibut (Fig. 4C). At the genus level, more genera were discriminative for Greenland halibut (Fig. 4D). These genera included the core genera *Pseudoalteromonas* and *Psychrobacter* or *Vibrio* and other genera, such as *Photobacterium*, *Burkholderia* and *Escherichia/Shigella.* The core genera *Staphylococcus* and *Acinetobacter* were discriminative exclusively for Atlantic halibut.

### Correlations with biotic and abiotic factors

**Figure 5:**
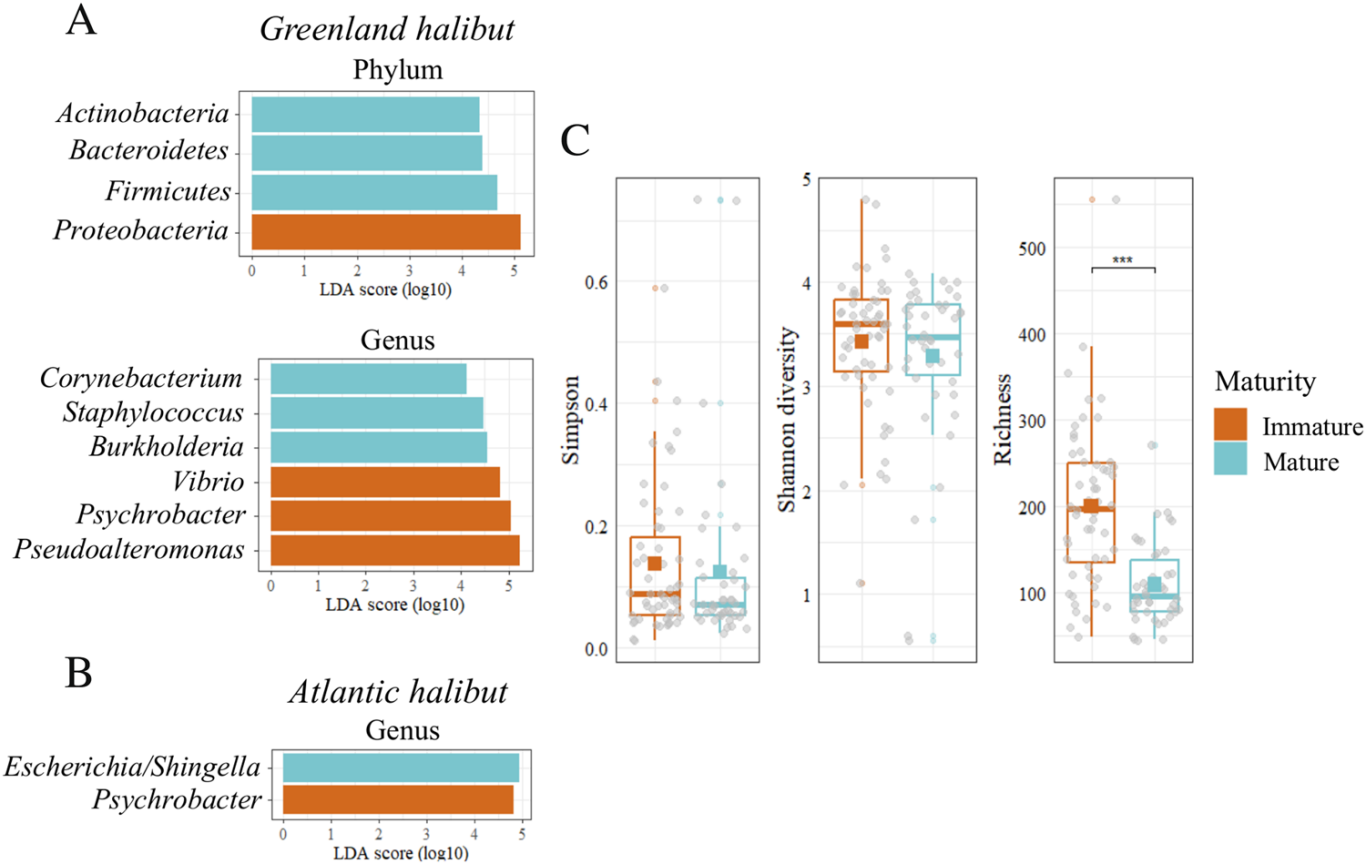
Maturity analysis. LEfSe analysis of the blood microbiome showing the significantly different taxa in mature (blue) and immature (red) fish in (**A**) Greenland halibut (Immature, n = 55, mature, n = 42) and (**B**) Atlantic halibut (Immature, n = 56, mature, n = 25). (**C**).α-Diversity metrics for immature and mature Greenland halibut. ***: *p* < 0.001.

Given the influence of the environment and the physiology on the microbiome, we next tested the hypothesis that the blood microbiome signature within each fish species varies according to different variables, including maturity classes (length), water temperature (measured at the sampling depth of the trawl), sex, and Fulton’s condition factor (K). We found that the blood microbiome structure varied depending on the maturity stage for both species at the phylum and genus levels. (Fig. 5A) In the case of Greenland halibut, significant differences in the abundance of *Corynebacterium*, *Staphylococcus*, *Burkholderia*, *Pseudoalteromonas*, *Psychrobacter* and *Vibrio* were noted (Fig. 5A, Figure S4). At the genus level, the most important difference between the mature and immature classes was in the case of *Vibrio*, which decreased by almost threefold in the mature class (from 9.7% to 3.3%; *p* < 0.001) (Table 3). The difference in *Burkholderia* between mature (0.7%) and immature (4.5%) Greenland halibut was also significant (*p* < 0.001). In the case of Atlantic halibut, the only two discriminative genera between both classes were *Psychrobacter* and *Escherichia/Shigella* (Fig. 5B, Figure S5). As for the α and β-diversity, some significant differences between the blood microbiome of mature and immature individuals were noted. In the case of Greenland halibut, we found a significant difference in the richness index (Fig. 5C), but no differences were noted for the Atlantic halibut (Figure S9). Significant differences in the β diversity between mature and immature individuals were found for both halibut species (Figures S6 and S7).

**Table 3:** Mean relative abundance of discriminative taxa found in the cmDNA in mature and immature halibuts.

Species	Taxa	Immature mean ± SE (n = 56)	Mature mean ± SE (n = 25)	*P* values	Test Statistics W
Greenland halibut	*Proteobacteria*	87.3 ± 1.1^*a*^	74.3 ± 2.8	< 0.001	620
	*Firmicutes*	5.1 ± 0.7	< 0.001	< 0.01	1543
	*Actinobacteria*	1.6 ± 0.4	3.8 ± 0.7	< 0.001	1615
	*Bacteroidetes*	3.3 ± 0.4	5.9 ± 0.9	< 0.05	1433
	*Vibrio*	9.7 ± 1.2	3.3 ± 0.9	< 0.001	438
	*Burkholderia*	0.7 ± 0.3	4.5 ± 0.9	< 0.001	1833
	*Corynebacterium*	0.4 ± 0.1	1.7 ± 0.5	< 0.001	1765
	*Psychrobacter*	21.1 ± 2.3	10.8 ± 2.0	< 0.001	670
	*Pseudoalteromonas*	31.5 ± 2.9	13.2 ± 2.2	< 0.001	521
	*Staphylococcus*	0.6 ± 0.2	3.6 ± 1.1	< 0.001	1676
Atlantic halibut	*Psychrobacter*	13.8 ± 2.0	7.9 ± 2.3	< 0.05	533
	*Escherichia/Shingella*	1.7 ± 1.5	9.5 ± 5.0	< 0.005	1008

^*a*^Values are expressed as percentages.

**Figure 6:**
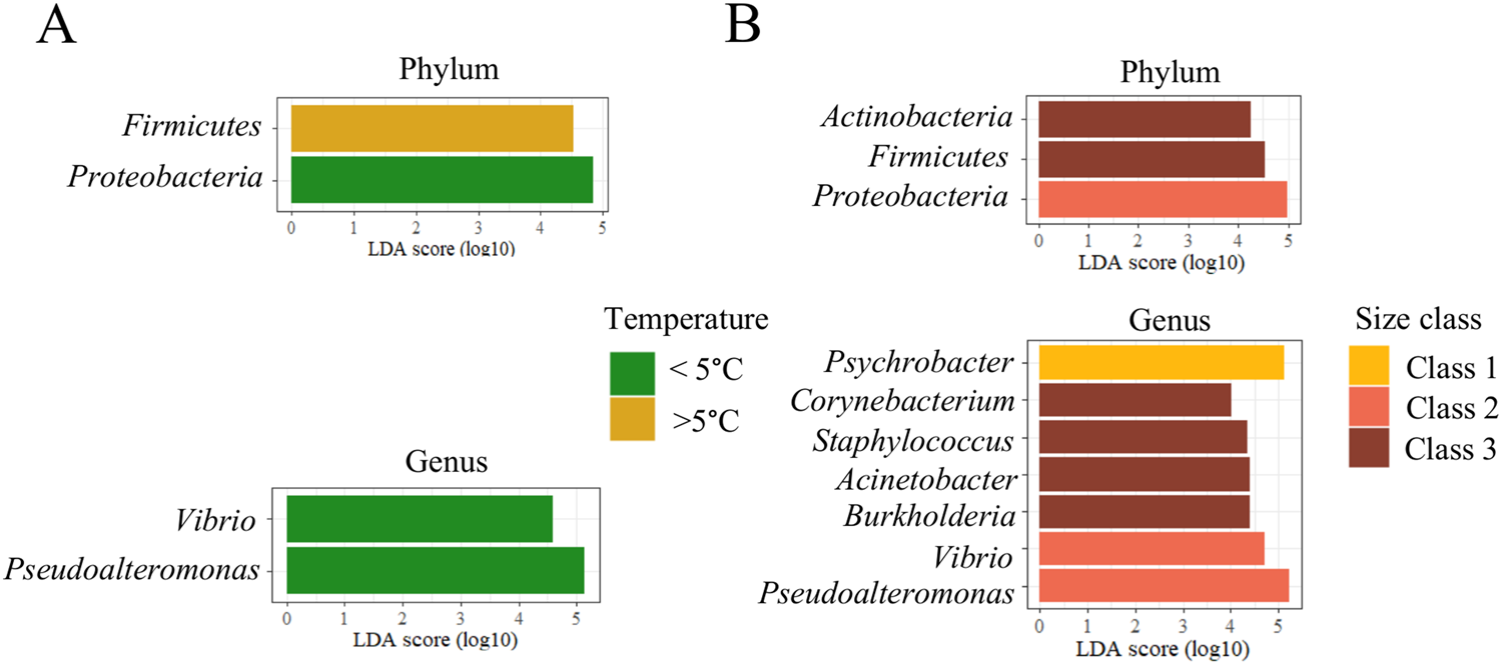
Discriminant taxa associated with seawater temperature and size classes. (**A**) LEfSe analysis of the blood microbiome showing the significantly different taxa in Greenland halibut (**A**) inhabiting cold (n = 25) and warm (n = 72) seawater and (**B**) according to their size class (Class 1, n = 36, Class 2, n = 22, Class 3, n = 39).

We next assessed whether temperature had an impact on the circulating microbiome signatures. Given the limited temperature tolerance of Greenland halibut^54^, we divided the populations into two groups based on whether they were sampled at temperatures below or above 5 °C. Atlantic halibuts have a wider temperature tolerance than the Greenland halibuts^45,46^, higher than the temperature recorded in this study. This is why no temperature analysis was carried out on this species. Our results showed significant temperature-related differences in Greenland halibut at both the phylum and genus levels (Fig. 6A, Figure S6). The relative abundance of *Pseudoalteromonas* was 34.4% in specimens distributed in relatively cold waters, compared to 19.9% in relatively warm water (*p* = 0.005) (Table 4). The relative abundance of *Vibrio* was also lower in Greenland halibut, distributed in warmer water (9.8%) than in cold water (5.9%) (*p* = 0.001). Similar findings were observed in the case of *Pseudoalteromonas* (Figure S8). Again, we found significant variations in the α and β diversity between temperature classes for Greenland halibut (Figures S6 and S9).

**Table 4:** Mean relative abundance (%) of discriminative taxa found in the cmDNA of Greenland halibuts inhabiting cold and warm water.

Taxa	Cold mean ± SE (n = 25)	Warm mean ± SE (n = 72)	*P* values	Test Statistics W
*Proteobacteria*	87.2 ± 2.0^*a*^	79.7 ± 1.9	< 0.05	626
*Firmicutes*	4.2 ± 0.8	7.9 ± 1.0	< 0.05	1166
*Pseudoalteromonas*	34.4 ± 4.9	19.9 ± 2.1	< 0.01	562
*Vibrio*	9.8 ± 1.4	5.9 ± 1.0	< 0.005	505.5

^*a*^Values are expressed as percentages.

We next determined whether the circulating microbiome varied in association with the size class of the Greenland halibut. Discriminative taxa included *Psychrobacter*, which was more abundant in fish-eating small prey (Fig. 6B), with the highest mean relative abundance of 27.9% in this group (Kruskal‒Wallis; *p* < 0.001) (Table 5). The two other core genera were identified as discriminative in intermediate-sized prey-eating fish, with a mean relative abundance of 10.4% for *Vibrio* (*p* < 0.001) and 37.2% for *Pseudoalteromonas* (*p* < 0.001). *Corynebacterium*, *Staphylococcus*, *Acinetobacter* and *Burkholderia* were discriminative for large prey-eating fish (Figure S10). All these genera were previously documented in teleost fishes' gut or skin microbiome, and *Acinetobacter* was identified as a biomarker of carnivorous fish (Huang et al. 2020).

**Table 5:** Mean relative abundance (%) of the discriminative taxa found in the cmDNA of Greenland halibuts (*R. hippoglossoides*) according to the size classes.

Taxa	Class 1 mean ± SE (n = 36)	Class 2 mean ± SE (n = 22)	Class 3 mean ± SE (n = 39)	*P* values^*b*^	Test Statistics Kruskal–Wallis χ^2^
*Proteobacteria*	86.4 ± 1.4	87.5 ± 2.0	73.9 ± 3.0	< 0.005	13.24
*Actinobacteria*	1.8 ± 0.5	1.6 ± 0.4	3.9 ± 0.7	< 0.01	10.252
*Firmicutes*	5.8 ± 1.0	4.5 ± 1.0	9.3 ± 1.6	< 0.05	6.5906
*Vibrio*	8.8 ± 1.6	10.4 ± 1.6	3.2 ± 1.0	< 0.001	26.687
*Psychrobacter*	27.9 ± 2.9	8.3 ± 1.4	10.8 ± 2.1	< 0.001	26.643
*Acinetobacter*	2.8 ± 0.7	1.4 ± 0.4	5.0 ± 1.2	< 0.05	6.7541
*Pseudolalteromonas*	27.0 ± 2.9	37.2 ± 5.7	12.9 ± 2.2	< 0.001	21.364
*Burkholderia*	0.8 ± 0.4	1.8 ± 0.9	4.1 ± 0.9	< 0.001	20.681
*Alcaligenes*	0.1 ± 0.1	1.4 ± 1.1	0.02 ± 0.01	< 0.001	21.464
*Corynebacterium*	0.4 ± 0.2	0.3 ± 0.1	1.7 ± 0.5	< 0.001	18.396
*Staphylococcus*	0.7 ± 0.2	0.8 ± 0.3	3.7 ± 1.2	< 0.001	14.658

^*a*^Values are expressed as percentages.

Concerning differences between males and females, we found only a few statistically significant differences (Figure S11). *Shewanella* and *Psychrobacter* were more abundant in Greenland and Atlantic male halibut, respectively, whereas the relative abundance of *Acinetobacter* was more abundant in Atlantic halibut females. This abundance of *Shewanella* in female versus male Greenland halibut was significant (0.4% in females compared to 3.2% in males). We did not observe any variations in the α and β diversity between males and females for either species (Figures S6, S7, and S9).

Finally, according to the factor K_rel_ conditions, a health index based on the individual’s plumpness, only *Photobacterium* was highlighted as a discriminative genus for low-condition Atlantic halibut. In contrast, Streptococcus was a marker of high-condition individuals (Figure S12). In Greenland halibut, the only significant difference was the lower abundance of *Escherichia/Shigella* in the Greenland halibut with a low relative condition factor (K).

### Spatial variation

**Figure 7:**
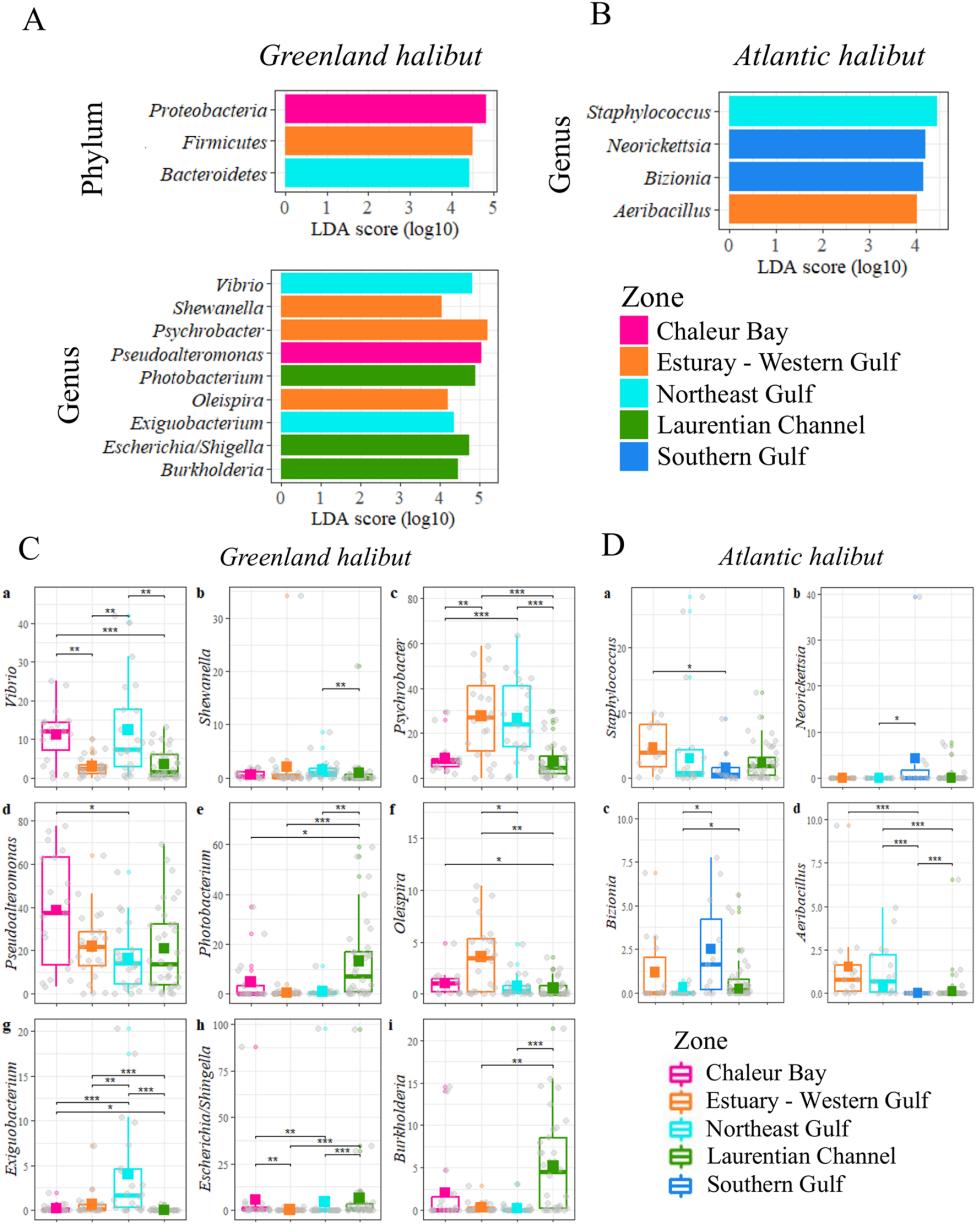
Spatial analysis. LEfSe analysis of the blood microbiome showing the significantly different taxa in (**A**) Greenland and (**B**) Atlantic halibut according to their localization. (**C** and **D**). Relative abundance of the discriminative genera in Greenland and Atlantic halibut. (*) *p* < 0.05; (**) p < 0.01; (***) *p* < 0.00. Greenland halibut: Estuary–Western Gulf, n = 22, Chaleur Bay, n = 19, Northeast Gulf, n = 22, Laurentian Channel, n = 34. Atlantic halibut: Estuary–Western Gulf, n = 14, Southern Gulf, n = 11, Northeast Gulf, n = 17, Laurentian Channel, n = 44.

We next tested the hypothesis that variations in the circulating microbiome could be attributed to geographic distribution. LEfSe analyses showed several variations at the genus level in both halibut species that were dependent on the collection site (Fig. 7A,B). More specifically, in the case of Greenland halibut, *Photobacterium* and *Burkholderia* were significantly more abundant in the Laurentian Channel than in the other areas with mean relative abundances of 13.2 ± 2.8% (*p* < 0.001) and 5.2 ± 1.0% (*p* < 0.001), respectively (Fig. 7C). *Exiguobacterium* and *Oleispira* were also more abundant in the Northeast Gulf (*p* < 0.001), whereas *Oleispira* was especially abundant in the Estuary and Western Gulf (*p* < 0.001). This finding contrasted with that noted for *Psychrobacter*, which was equally abundant in the Northeast gulf and the Estuary–Western gulf but less abundant in the Chaleur Bay and the Laurentian channel. Among other notable differences in Greenland halibut, we found a higher abundance of *Vibrio* in the Chaleur Bay and Northeast Gulf (*p* < 0.001). In the case of the Atlantic halibut, the Southern Gulf was characterized by a higher abundance of *Bizionia* (*p* = 0.009) and *Neorickettsia* (*p* = 0.015) relative to that in the Northeast Gulf and a lower abundance of *Staphylococcus* compared to the Estuary and Western Gulf (*p* = 0.039) (Fig. 7D). In terms of biodiversity, we did not find notable differences among areas except for a lower richness in the Laurentian Channel compared to Chaleur Bay (Figure S9).

## Discussion

The circulating microbiome is an emerging concept that has drawn a high level of interest in the biomedical field, given its potential to generate predictive biomarkers and the means to screen for potential pathogens. In the present work, we applied this concept to characterize the circulating microbiome signature to two wild halibut populations of economic and ecological importance. We further studied how the microbiome signature composition and structure correlate with physiological and environmental factors.

Typically, in humans and other species, including fish, most microbiome studies have focused on the gut microbiome. In this study, even though the fish were euthanized during the DFO bottom survey and sex was assessed by visual characterization, the individual’s sex could have been genetically determined with the blood samples^55,56^. In addition to ethical and logistical considerations, recent studies in humans and other species have shown that defining the core blood microbiome using a single drop of blood exhibits considerable potential as a disease biomarker^16,20^. The circulating microbiome can be used to facilitate the detection of pathogens, given that pathogenic (and symbiotic) bacteria are not exclusively found in the gut but also in other tissues. It is important to note, however, that the presence of bacteria in the blood is not de facto associated with a disease state, as the existence of a healthy blood microbiome is increasingly recognized^16^. Indeed, the paradigm that the blood is a sterile compartment has shifted radically since the development of 16S rRNA next-generation sequencing methods. However, it is not clear at present whether the blood microbiome reflects bacteria that inhabit the blood in dormancy^16^ or bacteria that translocate from one niche to another via blood circulation, a process referred to as “atopobiosis”^57^. According to this hypothesis, not only do bacteria use blood vessels to migrate from one tissue to another, but they can also do so by protecting themselves by infecting erythrocytes or white blood cells. Notwithstanding these fundamental questions, defining a dysbiotic blood microbiome has become a promising avenue for developing clinical biomarkers^58,59^. Combined with a logistically simple method based on a single drop of blood that can be stored at room temperature on cellulose paper, our study thus opens the door to developing a new type of biomarker that could be easily integrated into long-term monitoring programs.

From a fundamental point of view, our study has revealed that the blood microbiome of Greenland halibut and Atlantic halibut is unexpectedly richer than that reported to date in the blood of other endotherm animals^21–24^, as well as in the gut or skin microbiome of other fish and marine species^38,40,41,60–68^. In addition, we found close similarities with core blood microbiomes, from other species and organs, such as skin and gut microbiomes, in terms of phyla (*Proteobacteria*, *Firmicutes*, *Bacteroidetes* and *Actinobacteria*) and genera^12,41,69^. However, a first glimpse at the blood microbiome of these two species revealed that their core blood microbiomes share many features, most notably at the phylum and genus levels. Overall, 30 different phyla, 63 classes, and 685 genera were identified within the cmDNA of both species. At the phylum level, the microbiome signatures of the cmDNA were dominated by *Proteobacteria*, *Firmicutes*, *Bacteroidetes* and *Actinobacteria*, although both species showed significantly different relative abundances in their phyla. At the genus level, the aggregated core genera were dominated by *Pseudoalteromonas*, *Psychrobacter* and *Acinetobacter* for both fish. The core ASV analysis provides more information on the core bacteria found with the aggregated genera. While the core genera were based on hundreds of ASVs (ASVs that belong to the same genera were aggregated together), those ASV were genetically different from each other. A closer analysis of each ASV, approximately 30% (on average) of the microbiome was represented by only five ASVs for Atlantic halibut and nine for Greenland halibut out of 3362 and 5023 ASVs in total, respectively (Figure S13). For example, *Pseudoalteromonas* represented an average of 23.6% of the Greenland halibut circulating microbiome when considering all 132 ASVs characterized as *Pseudoalteromonas*. However, the mean relative abundance is unequal among those ASVs, given that one represents 18.2% on average and the other only 5.4%. Moreover, no *Vibrio* nor *Staphylococcus* ASV stood out in the ASV analysis, even though the prevalence was lower. This finding indicates that *Vibrio* and *Staphylococcus* vary considerably more among individuals than *Pseudoalteromonas*, *Psychrobacter* and *Acinetobacter*.

Another notable difference between fish circulating microbiomes was the α- and β-diversity, and the richness per individual was higher in Greenland halibut. This microbiome was also more diverse in terms of the number of ASVs (5023 ASVs against 3362 ASVs in Atlantic halibut). We also found distinct genera in each species (*Enhydrobacter* for the Atlantic halibut and *Oleispira*, *Burkholderia* or *Shewanella,* among others for the Greenland halibut). All these differences could reflect genetic factors and coevolution history, as confirmed in the case of the gut microbiome^12,13,41^. Some genera have been found in the microbiome of other fish. For example, *Enhydrobacter* was identified in the mucus microbiome of three freshwater species^67^ and present in at least 50% of the circulating microbiome of Atlantic halibut in the current study. *Shewanella* is another example found in high abundance in the gut microbiome of Atlantic mackerel^12^ and previously described as an indicator of piscivorous behavior^41^. The environment is another factor that plays a role in the structure of the circulating microbiome. Greenland halibut is a cold-water (1–4 °C) species, whereas Atlantic Halibut is characterized by a higher temperature tolerance (1–13 °C). Our study also revealed that the relative abundance of the core genera and phyla varied according to size and temperature for Greenland halibut. *Vibrio* and *Pseudoalteromonas* were identified as discriminative taxa for each environmental factor studied. *Vibrio* and *Pseudoalteromonas* were abundant in cold water and immature individuals, particularly for fish-eating intermediate-sized prey. Additionally, *Psychrobacter* was significantly more abundant in immature fish for both species, especially in Greenland halibut eating small prey and male Atlantic halibut. Finally, *Acinetobacter* varied according to sex in Atlantic halibut and with the size class in Greenland halibut. It is, however, important to consider confounding factors before drawing conclusions. For example, in our case, sexual dimorphism implies that males are smaller than females at the same age in both species. Overall, our results indicate that biotic and abiotic factors, similar to the fish gut microbiome, impact the core blood microbiome^12,13,69^.

An interesting finding was the difference in the blood core microbiota between males and females. Their microbiome did not differ in α- or β-diversity. However, LEfSe analysis pointed to a genus that discriminated between males and females in Greenland halibut. Specifically, *Shewanella* was more prevalent in male Greenland halibut, as confirmed by the change in relative abundance. This genus did not appear in any other LEfSe for other classes. In humans and animals, sex is among the most important factors that shape the gut microbiome^70^. In fish, such differences between males and females have been reported for the gut microbiome. Indeed, a study in three-spined stickleback and Eurasian perch showed that the diet-associated microbiota is sex-dependent^71^.

Our LEfSe analysis comparing the blood microbiome between condition classes showed that fish with high condition levels presented significantly more Streptococcus than those with low-condition individuals in Atlantic halibut. In contrast, *Photobacterium* and *Vibrionaceae* were highlighted as discriminative taxa for low-condition Atlantic halibut. Although both genera have been identified in symbionts of other flatfish gut microbiomes^72,73^, they also comprise well-known pathogenic species^74,75^. One must consider, however, that Fulton’s K varies with multiple factors, including age, sex, and seasons.

Finally, our results highlighted spatial differences in the circulating microbiome signatures, particularly evident at the genus level (Figures S14 and S15)*.* Several factors could explain such variations, including the location of nurseries for Greenland halibuts in some of these areas^51^. The genera correlated with the nurseries area corresponded to those correlated with the immature individuals, i.e., *Psychrobacter* and *Vibrio*. However, it is important to consider potential confounding effects that may play a role in these variations, most notably for *Burkholderia*, which was also found in the large fish that are more common in the Laurentian Channel. Although the reasons behind these variations remain unclear, our data provide the basis for further investigation into the role of specific biotic and abiotic factors and how changes in these factors impact the circulating microbiome of these species.

In conclusion, our study provides answers to critical research questions about (1) fundamental differences between both halibut species and (2) the identification of factors that impact their circulating microbiome signatures. Our manuscript further provides a logistically-friendly sampling method for future longitudinal studies of dysbiosis in response to environmental stress factors. Based on the use of a single drop of blood fixed on cellulose paper, we hypothesize that the logistically friendly method we used is particularly well adapted for long-term monitoring of fish populations, most notably in response to climate change and the increase in ocean temperatures. It is important to note, however, that precautions, such as proper aseptic method, the use of sterile equipment, and controls (including no template controls and blank FTA cards) are essential to minimize the impact of contaminants in the interpretation of the results. Proper aseptic cleaning methods, the use of sterile equipment, no template controls (including control FTA cards and adjacent punches on the sampled cards), as well as the use of laboratories, materials and equipment specifically dedicated to the preparation of DNA needed, are necessary to remain critical in the interpretation of the results With such measures, given the stability of blood DNA on cellulose papers, this approach is perfectly adapted for biobanking purposes, facilitating future spatiotemporal retrospective studies. This study brings a first glimpse of what a circulating microbiome in fish looks like, but many questions remain unanswered. The similarities with the gut microbiome were striking and should be investigated. Furthermore, sex, maturity, diet and health indicators influenced the microbiome, especially the core microbiome. However, studies in a more controlled environment with finer maturity assessments, health and diet are needed to confirm these observations. Such studies would also reveal the impact of the environment on the relationship between physiological factors and the circulating microbiome. Future studies at a finer taxonomic level combined with multi-omics analysis, including transcriptomics and metabolomics, are also needed to determine the major driving factors that shape the blood microbiome in these two species.

## Supplementary Information

Supplementary Information.

## Data Availability

The sequence data supporting this study's findings are available on the NCBI website at https://www.ncbi.nlm.nih.gov/bioproject/PRJNA853332. The full dataset is available at https://doi.org/10.6084/m9.figshare.21482325, and the Rstudio code is available at https://doi.org/10.6084/m9.figshare.21482355.

## Abbreviations

*R. hippoglossoides*: *Reinhardtius hippoglossoides*
*H. hippoglossus*: *Hippoglossus hippoglossus*
cmDNA: Circulating microbiome DNA
PCoA: Principal coordinate analysis
ASV: Amplicon sequence variants
OTU: Operational taxon unit
PERMANOVA: Multivariate analysis of variance with permutation
LEfSe: Linear discriminant analysis effect size

## References

[1] Carrier E, Ferchaud A, Normandeau E, Sirois P, Bernatchez L (2020). Estimating the contribution of Greenland Halibut (*Reinhardtius hippoglossoides*) stocks to nurseries by means of genotyping-by-sequencing: Sex and time matter. Evol. Appl..

[2] Kess T, Einfeldt AL, Wringe B, Lehnert SJ, Layton KK, McBride MC, Robert D, Fisher J, Le Bris A, den Heyer C, Shackell N (2021). A putative structural variant and environmental variation associated with genomic divergence across the Northwest Atlantic in Atlantic Halibut. ICES J. Mar. Sci..

[3] DFO. Assessment of the Gulf of St. Lawrence (4RST) Greenland halibut stock in 2020. *DFO Can. Sci. Advis. Sec.* Sci. Advis. Rep. 2021/017. (2021).

[4] DFO. Stock Assessment of Gulf of St. Lawrence (4RST) Atlantic Halibut in 2020. *DFO Can. Sci. Advis. Sec.* Sci. Advis. Rep. 2021/034. (2021).

[5] Shackell NL, Fisher JA, den Heyer CE, Hennen DR, Seitz AC, Le Bris A, Robert D, Kersula ME, Cadrin SX, McBride RS, McGuire CH (2022). Spatial ecology of Atlantic Halibut across the Northwest Atlantic: A recovering species in an era of climate change. Rev. Fisheries Sci. Aquac..

[6] Brown-Vuillemin S, Chabot D, Nozères C, Tremblay J, Sirois P, Robert D (2022). Diet composition of redfish (Sebastes sp) during periods of population collapse and massive resurgence in the Gulf of St. Lawrence. Front. Mar. Sci..

[7] Gauthier, J., Marquis, M.-C., Bourdages, H., Ouellette-Plante, J. & Nozères, C. Gulf of St. Lawrence (4RST) Greenland Halibut Stock Status in 2018: Commercial Fishery and Research Survey Data. *DFO Can. Sci. Advis. Sec.* **Res. Doc. 2020/016**, v +130 (2020).

[8] Gilbert JA, Quinn RA, Debelius J, Xu ZZ, Morton J, Garg N, Jansson JK, Dorrestein PC, Knight R (2016). Microbiome-wide association studies link dynamic microbial consortia to disease. Nature.

[9] Ghanbari M, Kneifel W, Domig KJ (2015). A new view of the fish gut microbiome: Advances from next-generation sequencing. Aquaculture.

[10] Suchodolski JS (2016). Diagnosis and interpretation of intestinal dysbiosis in dogs and cats. Vet. J..

[11] Bozzi D, Rasmussen JA, Carøe C, Sveier H, Nordøy K, Gilbert MTP, Limborg MT (2021). Salmon gut microbiota correlates with disease infection status: potential for monitoring health in farmed animals. Anim. Microbiome.

[12] Egerton S, Culloty S, Whooley J, Stanton C, Ross RP (2018). The gut microbiota of marine fish. Front. Microbiol..

[13] Legrand TPRA, Wynne JW, Weyrich LS, Oxley APA (2020). A microbial sea of possibilities: Current knowledge and prospects for an improved understanding of the fish microbiome. Rev. Aquacult..

[14] Perry WB, Lindsay E, Payne CJ, Brodie C, Kazlauskaite R (2020). The role of the gut microbiome in sustainable teleost aquaculture. Proc. R. Soc. B..

[15] Reverter M, Tapissier-Bontemps N, Lecchini D, Banaigs B, Sasal P (2018). Biological and ecological roles of external fish mucus: A review. Fishes.

[16] Castillo DJ, Rifkin RF, Cowan DA, Potgieter M (2019). The healthy human blood microbiome: Fact or fiction?. Front. Cell. Infect. Microbiol..

[17] Kowarsky M, Camunas-Soler J, Kertesz M, De Vlaminck I, Koh W, Pan W, Martin L, Neff NF, Okamoto J, Wong RJ, Kharbanda S, El-Sayed Y, Blumenfeld Y, Stevenson DK, Shaw GM, Wolfe ND, Quake SR (2017). Numerous uncharacterized and highly divergent microbes which colonize humans are revealed by circulating cell-free DNA. Proc. Natl. Acad. Sci. USA.

[18] Panaiotov S, Filevski G, Equestre M, Nikolova E, Kalfin R (2018). Cultural isolation and characteristics of the blood microbiome of healthy individuals. AIDS Patient Care STDs.

[19] Poore GD, Kopylova E, Zhu Q, Carpenter C, Fraraccio S, Wandro S, Janssen S, Metcalf J, Song SJ, Kanbar J, Miller-Montgomery S, Haeton R, Mckay R, Patel SP, Swafford AD, Knight R (2020). Microbiome analyses of blood and tissues suggest cancer diagnostic approach. Nature.

[20] Whittle E, Leonard MO, Harrison R, Gant TW, Tonge DP (2019). Multi-method characterization of the human circulating microbiome. Front. Microbiol..

[21] Mandal RK, Jiang T, Al-Rubaye AA, Rhoads D, Wideman RF, Zhao J, Pevzner I, Kwon YM (2016). An investigation into blood microbiota and its potential association with Bacterial Chondronecrosis with Osteomyelitis (BCO) in Broilers. Sci. Rep..

[22] Scarsella E, Sandri M, Monego SD, Licastro D, Stefanon B (2020). Blood microbiome: A new marker of gut microbial population in dogs?. Vet. Sci..

[23] Scarsella E, Zecconi A, Cintio M, Stefanon B (2021). Characterization of microbiome on feces, blood and milk in dairy cows with different milk leucocyte pattern. Animals.

[24] Vientós-Plotts AI, Ericsson AC, Rindt H, Grobman ME, Graham A, Bishop K, Cohn LA, Reinero CR (2017). Dynamic changes of the respiratory microbiota and its relationship to fecal and blood microbiota in healthy young cats. PLoS ONE.

[25] Amar J, Lange C, Payros G, Garret C, Chabo C, Lantieri O, Courtney M, Marre M, Charles MA, Balkau B, Burcelin R (2013). Blood microbiota dysbiosis is associated with the onset of cardiovascular events in a large general population: The D.E.S.I.R. study. PLoS ONE.

[26] Hyun H, Lee MS, Park I, Ko HS, Yun S, Jang D-H, Kim S, Kim H, Kang JH, Lee JH, Kwon T (2021). Analysis of porcine model of fecal-induced peritonitis reveals the tropism of blood microbiome. Front. Cell. Infect. Microbiol..

[27] Mohamed WMA, Ali AO, Mahmoud HYAH, Omar MA, Chatanga E, Salim B, Naguib D, Anders JL, Nonaka N, Moustafa MAM, Nakao R (2021). Exploring prokaryotic and eukaryotic microbiomes helps in detecting tick-borne infectious agents in the blood of camels. Pathogens.

[28] Tilahun Y, Pinango JQ, Johnson F, Lett C, Smith K, Gipson T, McCallum M, Hoyt P, Tritt A, Yadav A, Elshahed M, Wang Z (2022). Transcript and blood-microbiome analysis towards a blood diagnostic tool for goats affected by *Haemonchus contortus*. Sci. Rep..

[29] Vientós-Plotts AI, Ericsson AC, Rindt H, Reinero CR (2021). Blood cultures and blood microbiota analysis as surrogates for bronchoalveolar lavage fluid analysis in dogs with bacterial pneumonia. BMC Vet. Res..

[30] McMurdie PJ, Holmes S (2013). phyloseq: An R package for reproducible interactive analysis and graphics of microbiome census data. PLoS ONE.

[31] Ssekagiri, A., Ijaz, D. U. Z. & Sloan, W. T. MicrobiomeSeq: An R package for analysis of microbial communities in an environmental context. In *ISCB Africa ASBCB Conference* (Kumasi, Ghana, 2017).

[32] Cao, Y. *MicrobiomeMarker: Microbiome Biomarker Analysis. R Package Version 0.0. 1.9000*. Available online: https://github.com/yiluheihei/microbiomeMarker.

[33] Oksanen J, Blanchet FG, Friendly M, Kindt R, Legendre P, McGlinn D, Minchin PR, O’Hara RB, Simpson GL, Solymos P, Stevens MHH, Szoecs E, Wagnerm vegan H (2020). Community Ecology Package. R package version.

[34] Wickham H (2016). ggplot2: Elegant Graphics for Data Analysis.

[35] South, A. *Rnaturalearth: World Map Data from Natural Earth. R package version 0.1.0*. https://CRAN.R-project.org/package=rnaturalearth (2017).

[36] Palanisamy V, Gajendiran V, Mani K (2022). Meta-analysis to identify the core microbiome in diverse wastewater. Int. J. Environ. Sci. Technol..

[37] Jing H, Xiao X, Zhang Y, Li Z, Jian H, Luo Y, Han Z (2022). Composition and ecological roles of the core microbiome along the Abyssal-Hadal transition zone sediments of the Mariana trench. Microbiol. Spectr..

[38] Lokesh J, Kiron V (2016). Transition from freshwater to seawater reshapes the skin-associated microbiota of Atlantic salmon. Sci. Rep..

[39] Wang Y, Gong J, Li J, Xin Y, Hao Z, Chen C, Li H, Wang B, Ding M, Li W, Zhang Z, Xu P, Xu T, Ding G-C, Li J (2020). Insights into bacterial diversity in compost: Core microbiome and prevalence of potential pathogenic bacteria. Sci. Total Environ..

[40] Gatesoupe F-J, Zambonino Infante J-L, Cahu C, Quazuguek P (2016). The highly variable microbiota associated to intestinal mucosa correlates with growth and hypoxia resistance of sea bass, *Dicentrarchus labrax*, submitted to different nutritional histories. BMC Microbiol..

[41] Huang Q, Sham RC, Deng Y, Mao Y, Wang C, Zhang T, Leung KMY (2020). Diversity of gut microbiomes in marine fishes is shaped by host-related factors. Mol. Ecol..

[42] Huse SM, Ye Y, Zhou Y, Fodor AA (2012). A core human microbiome as viewed through 16S rRNA sequence clusters. PLoS ONE.

[43] DFO. Stock assessment of Gulf of St. Lawrence (4RST) Atlantic halibut in 2018. *DFO Can. Sci. Advis. Sec.* Sci. Advis. Rep. 2019/038 (2019).

[44] DFO. Assessment of the Gulf of St. Lawrence (4RST) Greenland Halibut stock in 2018. *DFO Can. Sci. Advis. Sec.* Sci. Advis. Rep. 2019/023 (2019).

[45] Jonassen TM, Imsland AK, Kadowaki S, Stefansson SO (2000). Interaction of temperature and photoperiod on growth of Atlantic halibut *Hippoglossus hippoglossus* L. Aquac. Res..

[46] Björnsson B, Tryggvadóttir SV (1996). Effects of size on optimal temperature for growth and growth efficiency of immature Atlantic halibut (*Hippoglossus hippoglossus* L.). Aquaculture.

[47] Le Cren ED (1951). The length-weight relationship and seasonal cycle in gonad weight and condition in the perch (*Perca fluviatilis*). J. Anim. Ecol..

[48] Froese R (2006). Cube law, condition factor and weight-length relationships: History, meta-analysis and recommendations. J. Appl. Ichthyol..

[49] Keys AB (1928). The weight-length relation in fishes. Proc. Natl. Acad. Sci. U.S.A..

[50] Blackwell BG, Brown ML, Willis DW (2000). Relative weight (Wr) status and current use in fisheries assessment and management. Rev. Fish. Sci..

[51] Youcef WA, Lambert Y, Audet C (2013). Spatial distribution of Greenland halibut Reinhardtius hippoglossoides in relation to abundance and hypoxia in the estuary and Gulf of St. Lawrence: *Greenland halibut distribution in estuary and Gulf of St Lawrence*. Fish. Oceanogr..

[52] Lahti, L., *et al*. *Microbiome R Package.* https://github.com/microbiome/microbiome/ (2018).

[53] Rstudio Team. RStudio: Integrated Development Environment for R. (2021).

[54] Morgan MJ, Garabana D, Rideout RM, Román E, Pérez-Rodríguez A, Saborido-Rey F (2013). Changes in distribution of Greenland halibut in a varying environment. ICES J. Mar. Sci..

[55] Ferchaud A-L, Mérot C, Normandeau E, Ragoussis J, Babin C, Djambazian H, Bérubé P, Audet C, Treble M, Walkusz W, Bernatchez L (2022). Chromosome-level assembly reveals a putative Y-autosomal fusion in the sex determination system of the Greenland Halibut (*Reinhardtius hippoglossoides*). G3 Genes Genomes Genet..

[56] Edvardsen RB, Wallerman O, Furmanek T, Kleppe L, Jern P, Wallberg A, Kjærner-Semb E, Mæhle S, Olausson SK, Sundström E, Harboe T, Mangor-Jensen R, Møgster M, Perrichon P, Norberg B, Rubin C-J (2022). Heterochiasmy and the establishment of gsdf as a novel sex determining gene in Atlantic halibut. PLoS Genet.

[57] Potgieter M, Bester J, Kell DB, Pretorius E (2015). The dormant blood microbiome in chronic, inflammatory diseases. FEMS Microbiol. Rev..

[58] Chen H, Ma Y, Liu Z, Li J, Li X, Yang F, Qiu M (2021). Circulating microbiome DNA: An emerging paradigm for cancer liquid biopsy. Cancer Lett..

[59] Wang C, Li Q, Tang C, Zhao X, He Q, Tang X, Ren J (2021). Characterization of the blood and neutrophil-specific microbiomes and exploration of potential bacterial biomarkers for sepsis in surgical patients. Immun. Inflamm. Dis..

[60] Bhute SS, Escobedo B, Haider M, Mekonen Y, Ferrer D, Hillyard SD, Friel AD, van Breukelen F, Hedlund BP (2020). The gut microbiome and its potential role in paradoxical anaerobism in pupfishes of the Mojave Desert. Anim. Microbiome.

[61] Etyemez M, Balcázar JL (2015). Bacterial community structure in the intestinal ecosystem of rainbow trout (*Oncorhynchus mykiss*) as revealed by pyrosequencing-based analysis of 16S rRNA genes. Res. Vet. Sci..

[62] Lowrey L, Woodhams DC, Tacchi L, Salinas I (2015). Topographical mapping of the rainbow trout (*Oncorhynchus mykiss*) microbiome reveals a diverse bacterial community with antifungal properties in the skin. Appl. Environ. Microbiol..

[63] Lyons PP, Turnbull JF, Dawson KA, Crumlish M (2017). Phylogenetic and functional characterization of the distal intestinal microbiome of rainbow trout *Oncorhynchus mykiss* from both farm and aquarium settings. J. Appl. Microbiol..

[64] Nielsen S, Walburn JW, Vergés A, Thomas T, Egan S (2017). Microbiome patterns across the gastrointestinal tract of the rabbitfish *Siganus fuscescens*. PeerJ..

[65] Reinhart EM, Korry BJ, Rowan-Nash AD, Belenky P (2019). Defining the distinct skin and gut microbiomes of the northern pike (*Esox lucius*). Front. Microbiol..

[66] Rosado D, Pérez-Losada M, Severino R, Cable J, Xavier R (2019). Characterization of the skin and gill microbiomes of the farmed seabass (*Dicentrarchus labrax*) and seabream (*Sparus aurata*). Aquaculture.

[67] Sylvain F-É, Holland A, Bouslama S, Audet-Gilbert É, Lavoie C, Val AL, Derome N (2020). Fish skin and gut microbiomes show contrasting signatures of host species and habitat. Appl. Environ. Microbiol..

[68] Walter JM, Bagi A, Pampanin DM (2019). Insights into the potential of the atlantic cod gut microbiome as biomarker of oil contamination in the marine environment. Microorganisms.

[69] Clements KD, Angert ER, Montgomery L, Choat JH (2014). Intestinal microbiota in fishes: What’s known and what’s not. Mol. Ecol..

[70] Kim YS, Unno T, Kim B-Y, Park M-S (2020). Sex differences in gut microbiota. World J. Mens. Health.

[71] Bolnick DI, Snowberg LK, Hirsch PE, Lauber CL, Org E, Parks B, Lusis AJ, Knight R, Caporaso JG, Svanbäck R (2014). Individual diet has sex-dependent effects on vertebrate gut microbiota. Nat. Commun..

[72] Cerdà-Cuéllar M, Blanch AR (2004). Determination of *Vibrio scophthalmi* and its phenotypic diversity in turbot larvae. Environ. Microbiol..

[73] Sugita H, Ito Y (2006). Identification of intestınal bacteria from Japanese flounder (*Paralichthys olivaceus*) and their ability to digest chitin. Lett. Appl. Microbiol..

[74] Thyssen A, Ollevier F, Whitman WB (2015). *Photobacterium*. Bergey’s Manual of Systematics of Archaea and Bacteria.

[75] Yeung PSM, Boor KJ (2004). Epidemiology, Pathogenesis, and Prevention of Foodborne *Vibrio parahaemolyticus* Infections. Foodborne Pathog. Dis..

