# Supplementary material for: Insights into the circulating microbiome of Atlantic and Greenland halibut populations: the role of species-specific and environmental factors
Source: Sci Rep. 2023 Apr 12;13:5971. doi: 10.1038/s41598-023-32690-6 (PMC10097863; doi:10.1038/s41598-023-32690-6)
Supplement: Supplementary file 1 — Supplementary Information. [file 41598_2023_32690_MOESM1_ESM.pdf]

SUPPLEMENTARY MATERIAL

Defining the circulating microbiome of wild-fish halibut populations using a single drop of blood: a novel approach to monitoring the health status of wild fish populations.

Fanny Fronton[1], Sophia Ferchiou[1], France Caza[1], Richard Villemur[1], Dominique Robert[2], and Yves St-Pierre[1].

1) INRS-Centre Armand-Frappier Santé Technologie, 531 Boul. des Prairies, Laval, QC, Canada, H7V 1B7

2) Institut des Sciences de la Mer, Université du Québec à Rimouski, 310, allée des Ursulines, C.P. 3300 Rimouski (Québec)

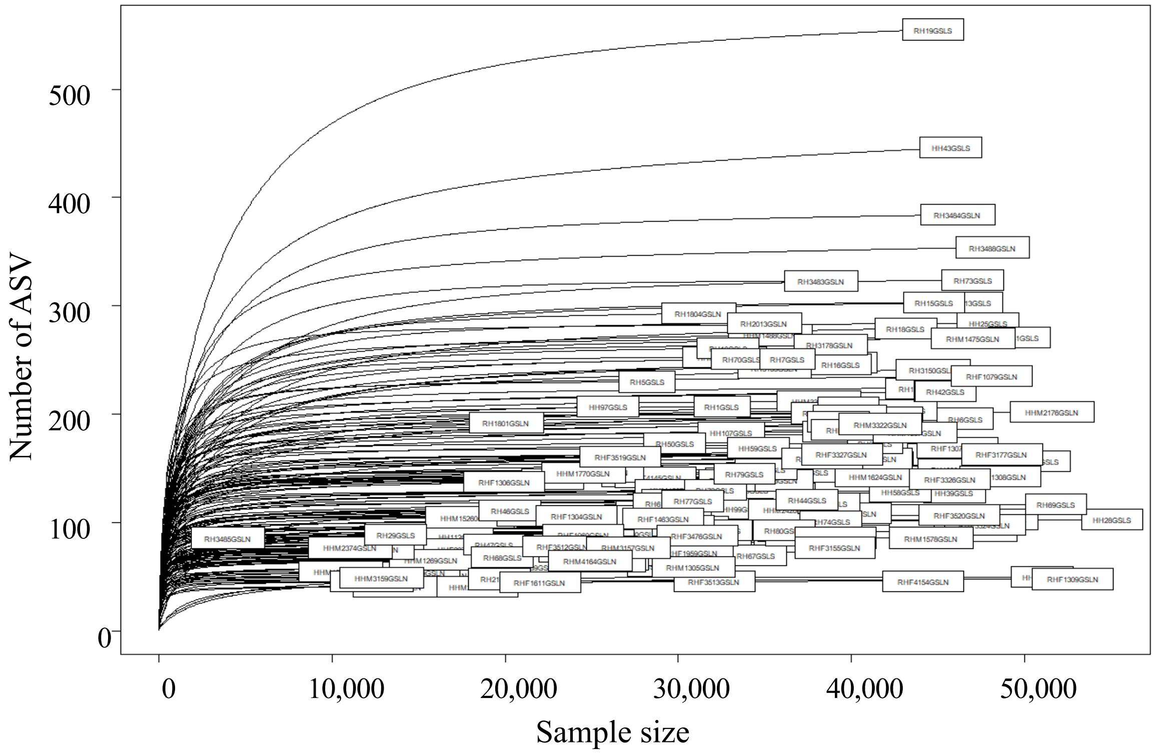

Figure S1: Sequencing depth curves based on the number of reads (sample size) and the number of ASVs for each sample. Each line represents one sample.

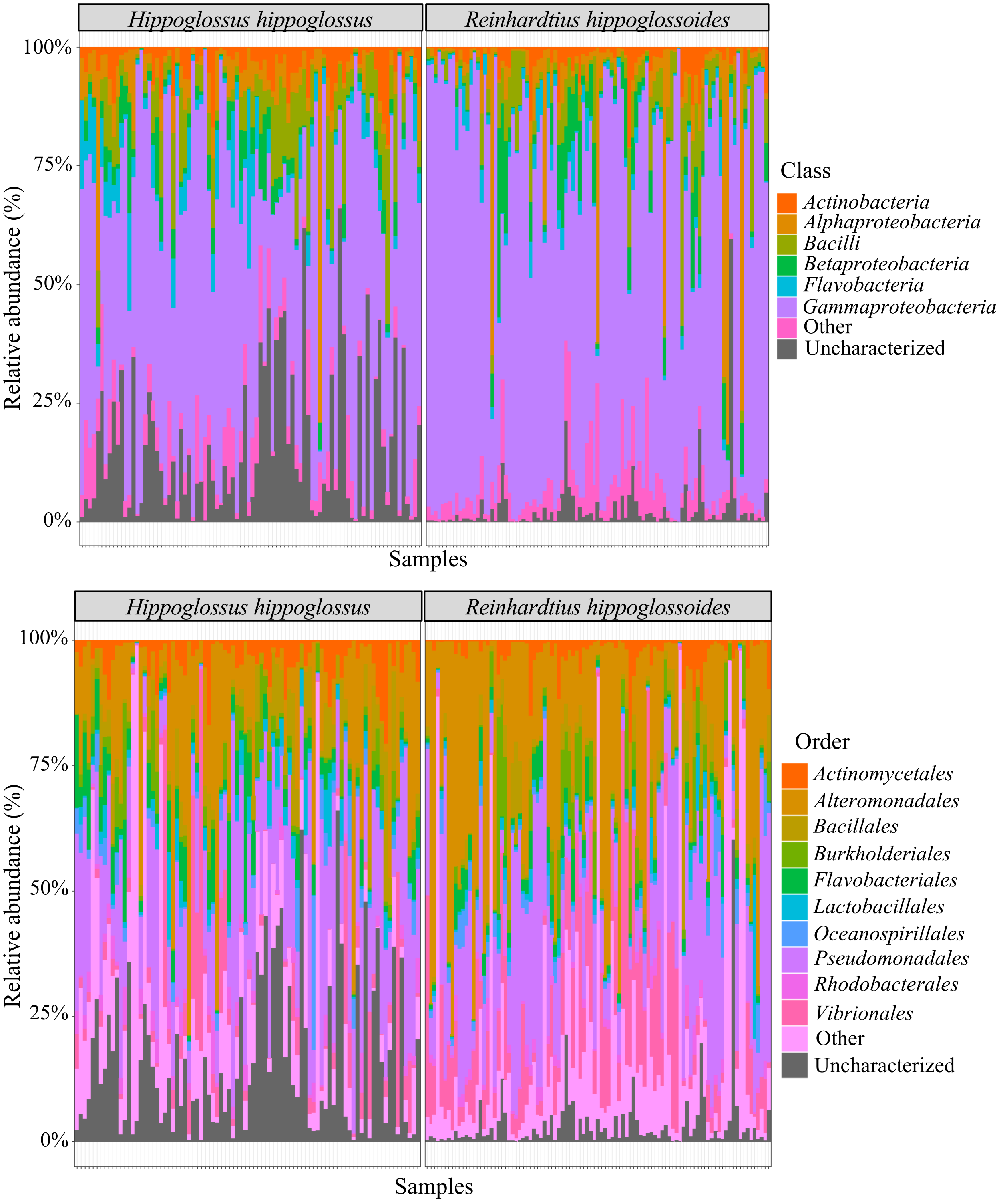

Figure S2: Relative abundance at the class and order levels of the halibuts' circulating microbiome. Relative abundance of the main classes (top) and orders (bottom) present the blood microbiome of the Atlantic halibut (left) and the Greenland halibut (right). Atlantic halibut, n = 86, Greenland halibut, n = 97.

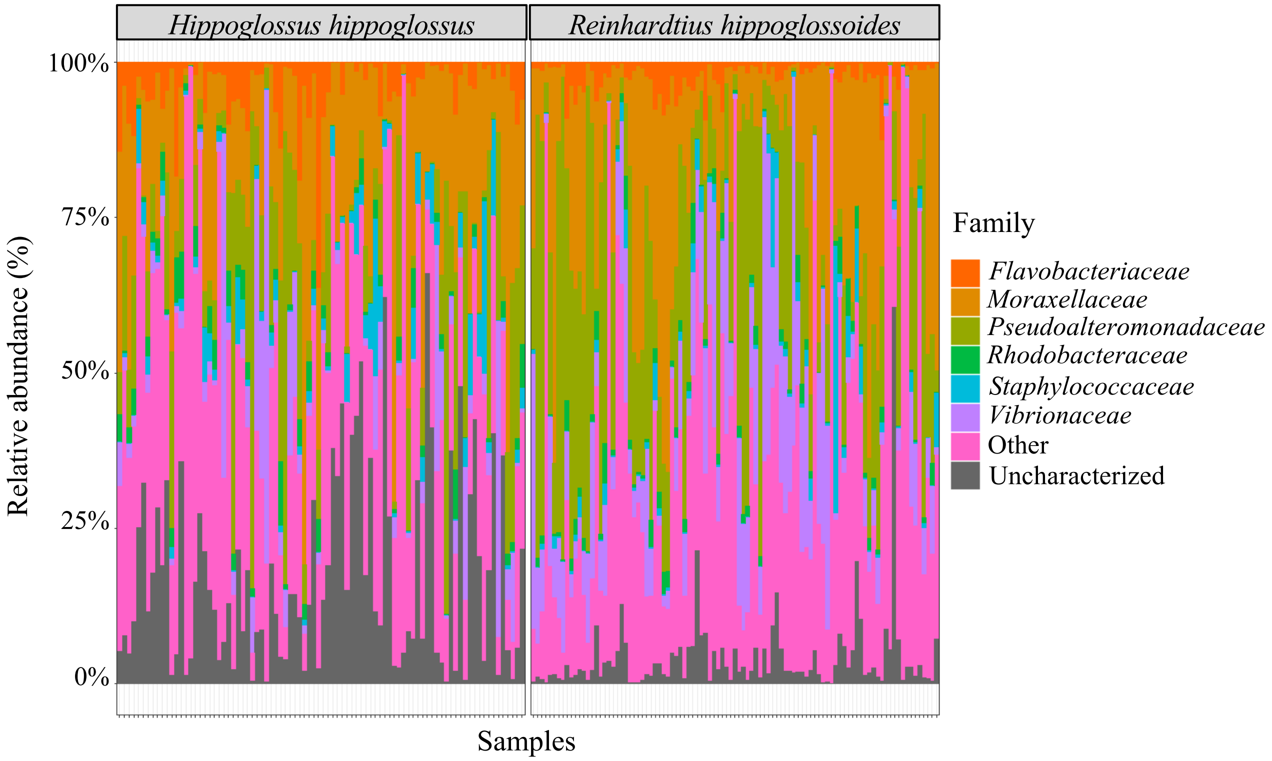

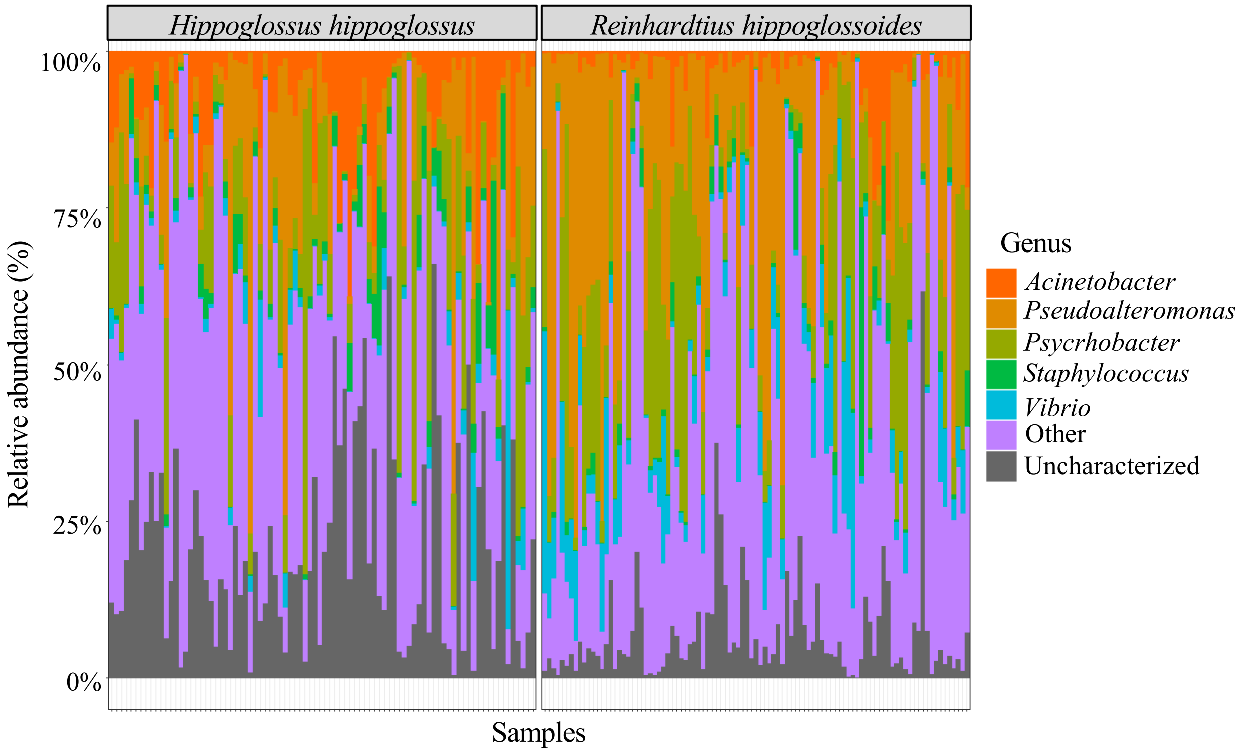

Figure S3: Relative abundance at the family and genus levels of the halibuts' circulating microbiome. Relative abundance of the main family (top) and genus (bottom) present the blood microbiome of the Atlantic halibut (left) and the Greenland halibut (right). Atlantic halibut, n = 86, Greenland halibut, n = 97.

Reinhardtius hippoglossoides

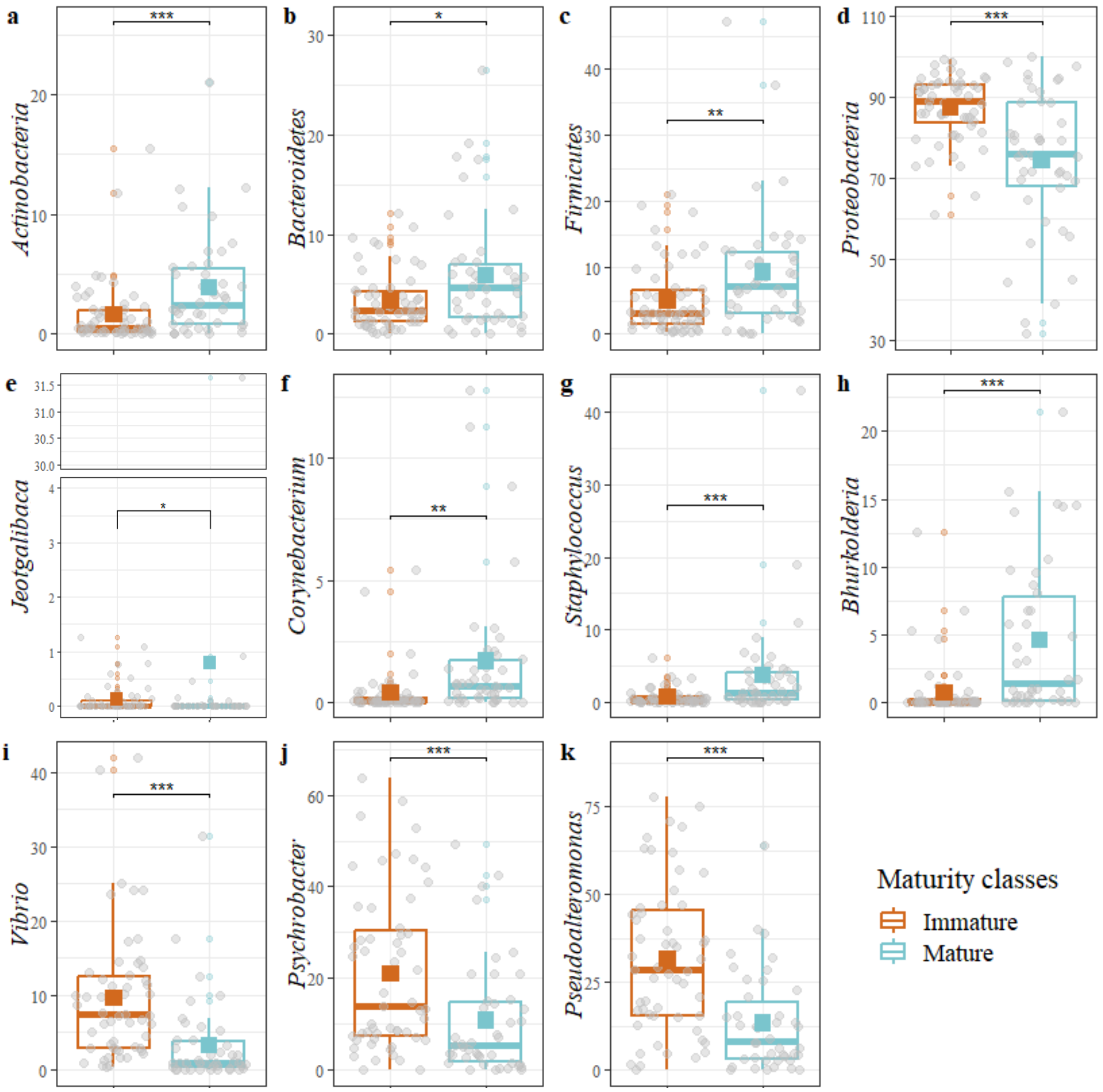

Figure S4: Relative abundance (%) of the discriminative phylum or genera found in the blood microbiome of immature (n = 55) and mature (n = 42) Greenland halibuts. (*) p < 0.05; (**) p < 0.01; (***) p < 0.001.

Hippoglossus hippoglossus

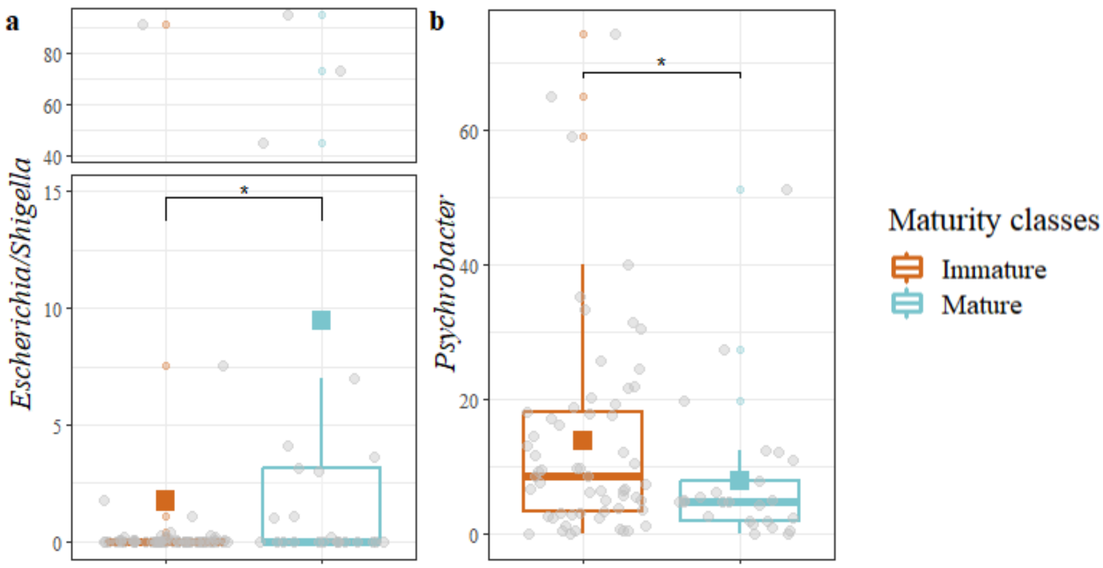

Figure S5: Relative abundance (%) of the discriminative phylum or genera found in the blood microbiome of immature (n = 56) and mature (n = 25) Atlantic halibut. (*) $p < 0.05$; (**) $p < 0.01$; (***) $p < 0.001$.

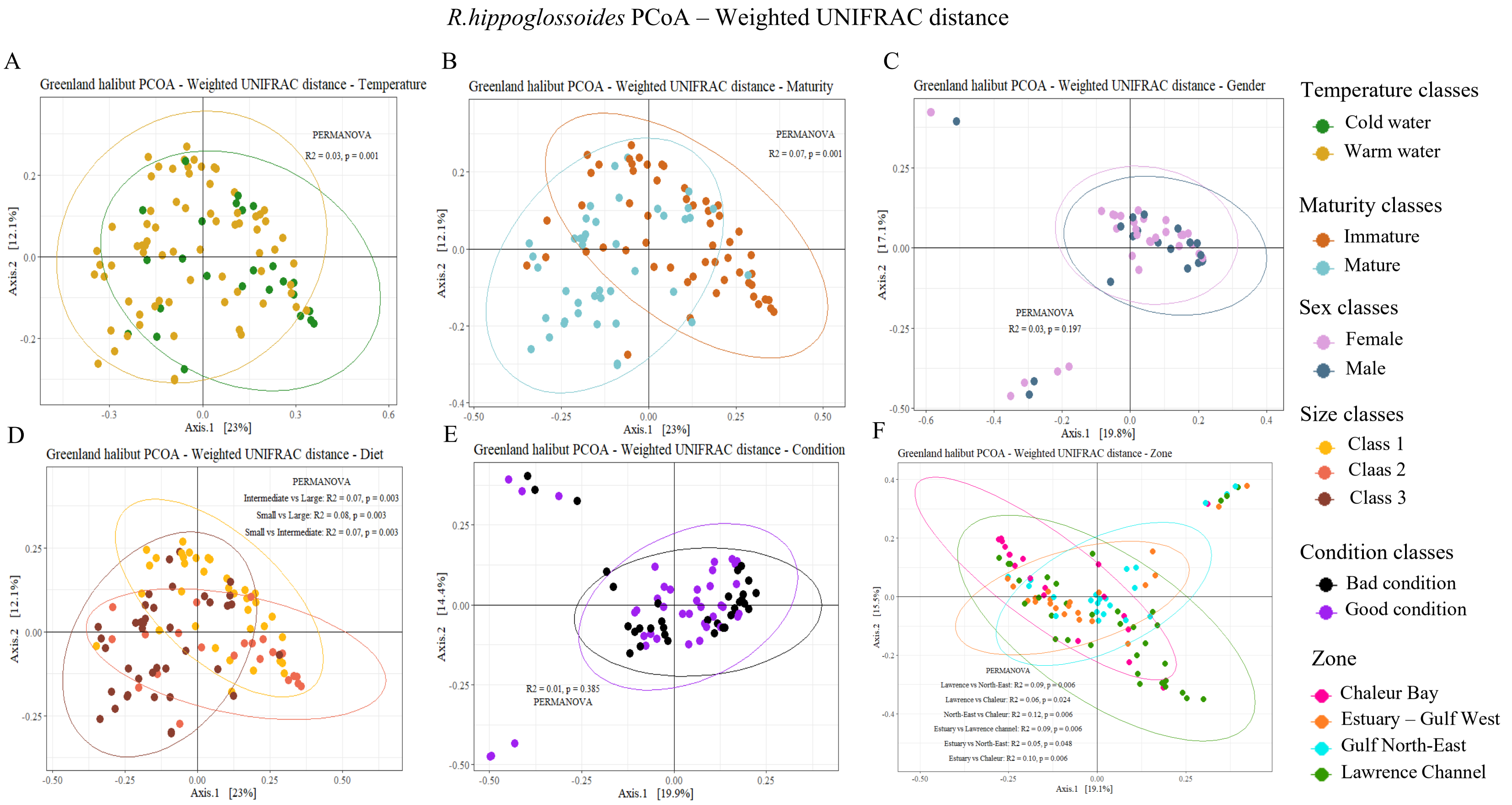

Figure S6: **PCoA (β-diversity) of the blood microbiome of the Greenland halibut (*R. hippoglossoides*).** The β-diversity was compared between temperature, diet, condition and maturity classes, sex, and zones.

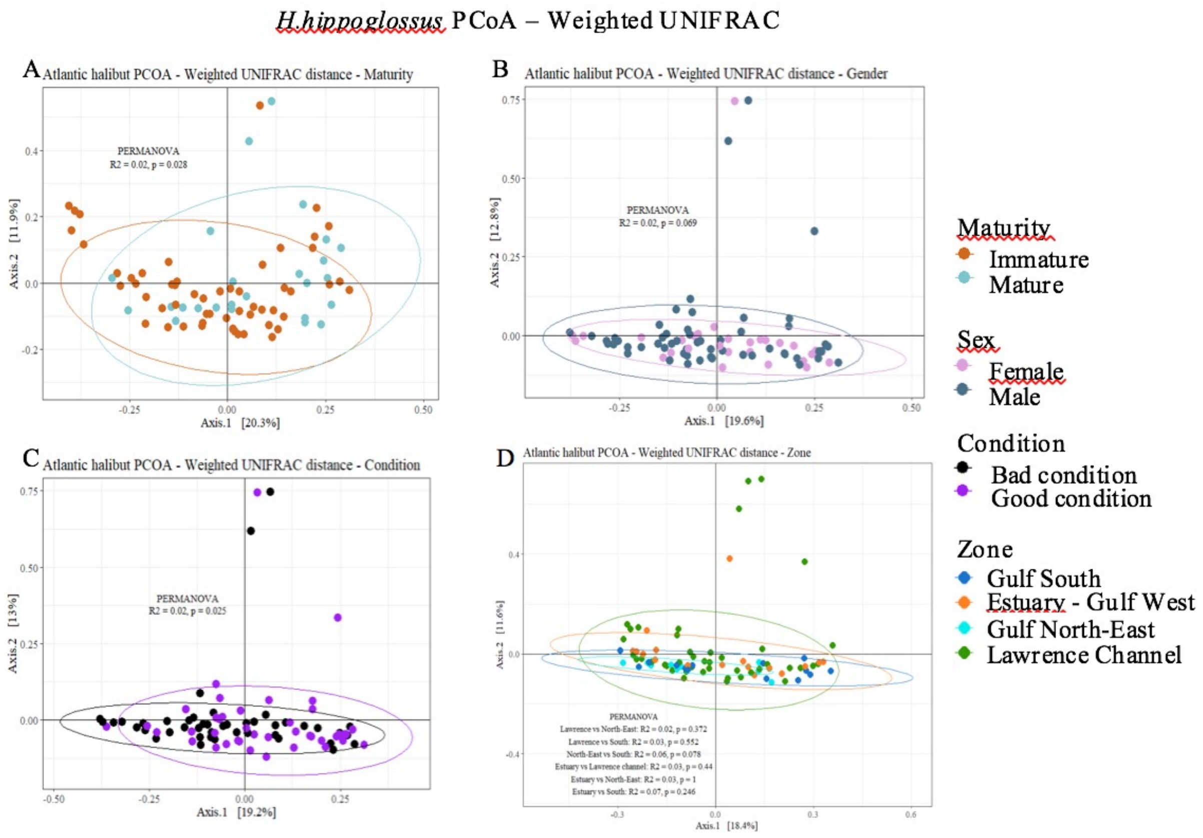

Figure S7: **PCoA (β-diversity) of the blood microbiome of the Atlantic halibut (*R. hippoglossus*).** The β-diversity was compared between condition and maturity classes, sex, and zones.

Reinhardtius hippoglossoides

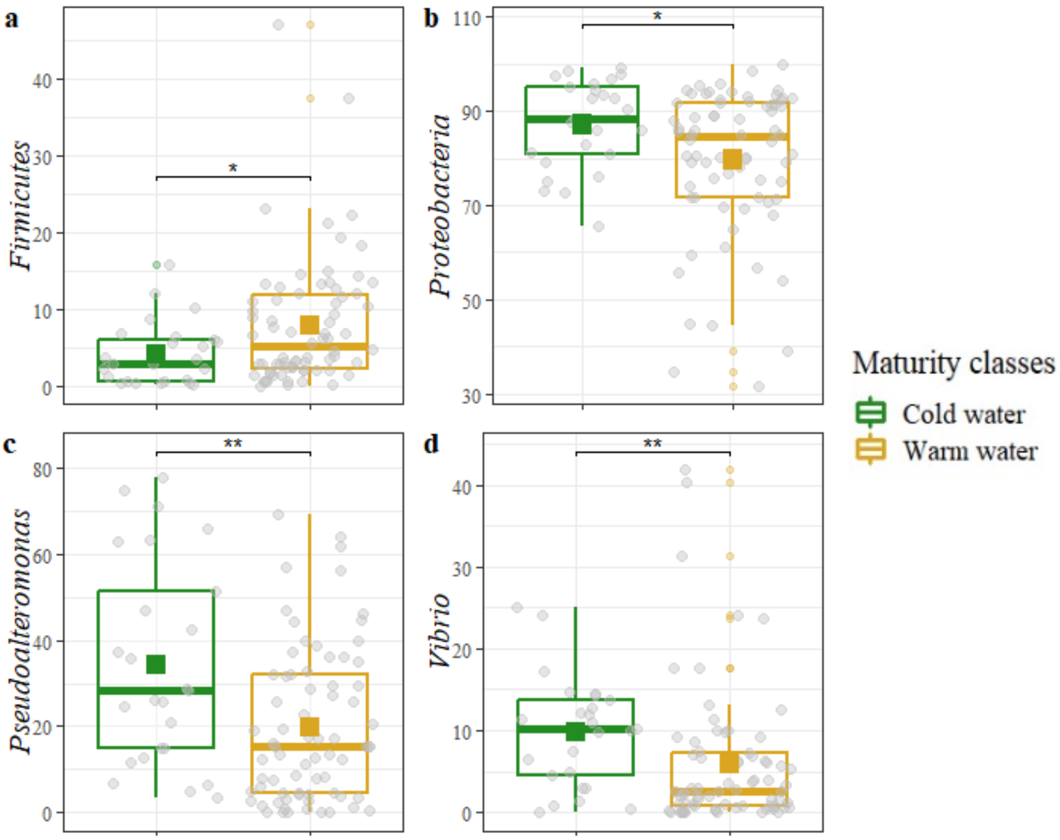

Figure S8: Relative abundance (%) of the discriminative phylum or genera in the blood of individuals inhabiting cold (<5°C) or warm (>5°C) water. Cold water, n = 25, warm water, n = 72. Significant differences between temperature classes were measured using the Wilcoxon-Mann-Whitney test. (*) $p < 0.05$; (**) $p < 0.01$; (***) $p < 0.001$.

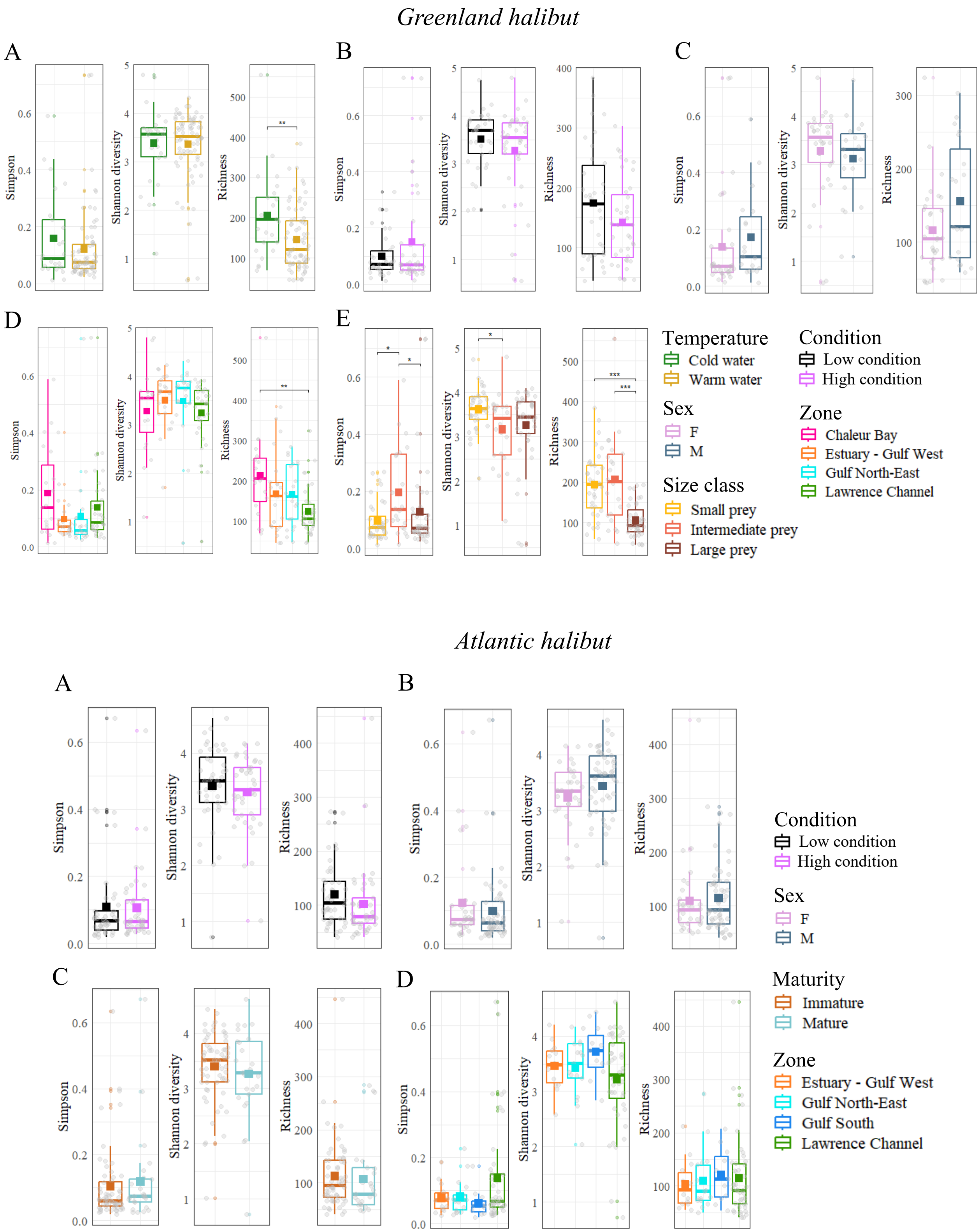

Figure S9: **Variations of α-diversity analysis of the blood microbiome of the Greenland and Atlantic halibut**.

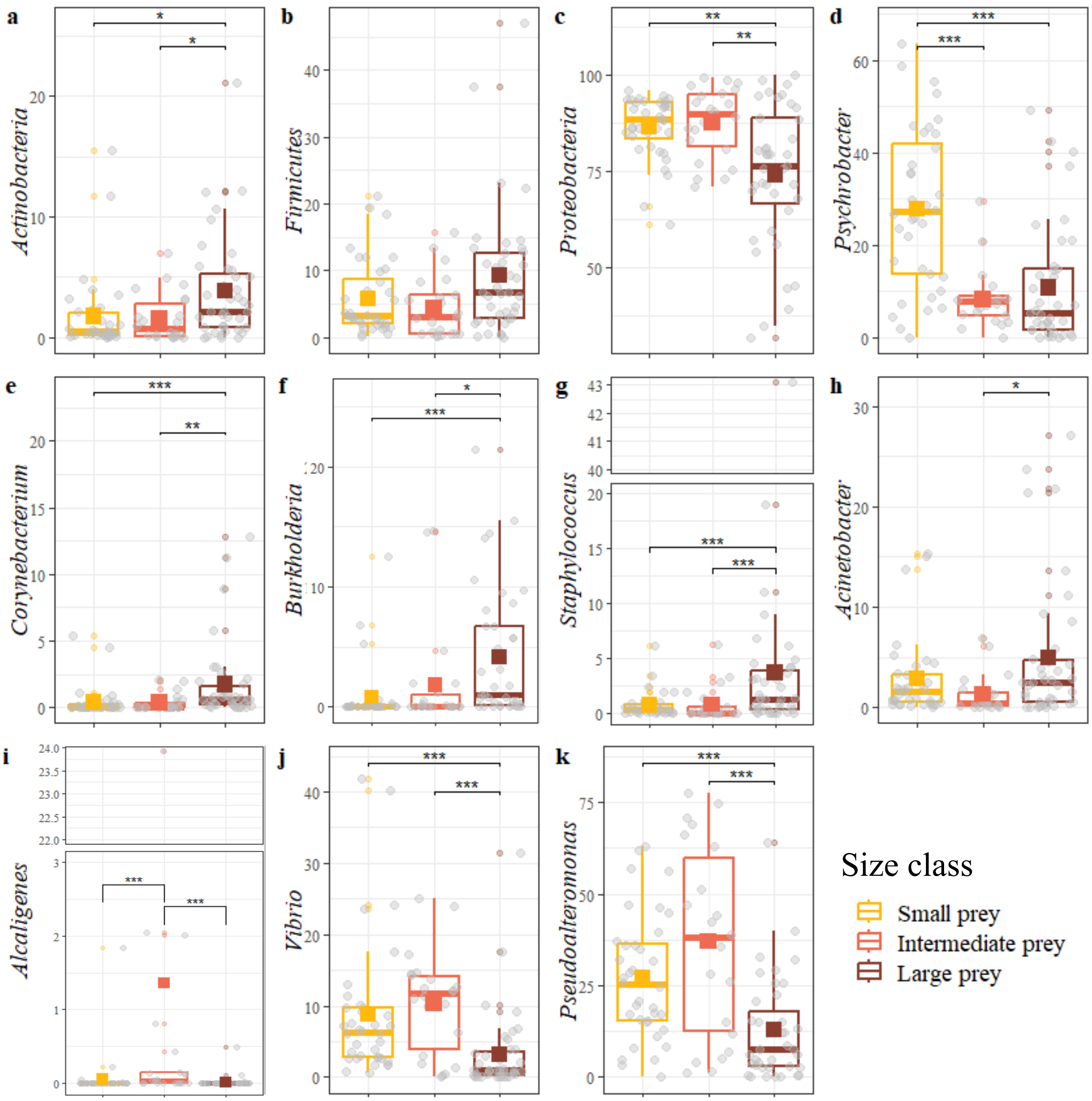

Figure S10: Relative abundance (%) of the discriminative phylum or genera in the blood of individuals according to diet classes. Small, n = 36, intermediate, n = 22, large, n = 39. Significant differences between temperature classes were measured using the Wilcoxon-Mann-Whitney test. (*) $p < 0.05$; (**) $p < 0.01$; (***) $p < 0.001$.

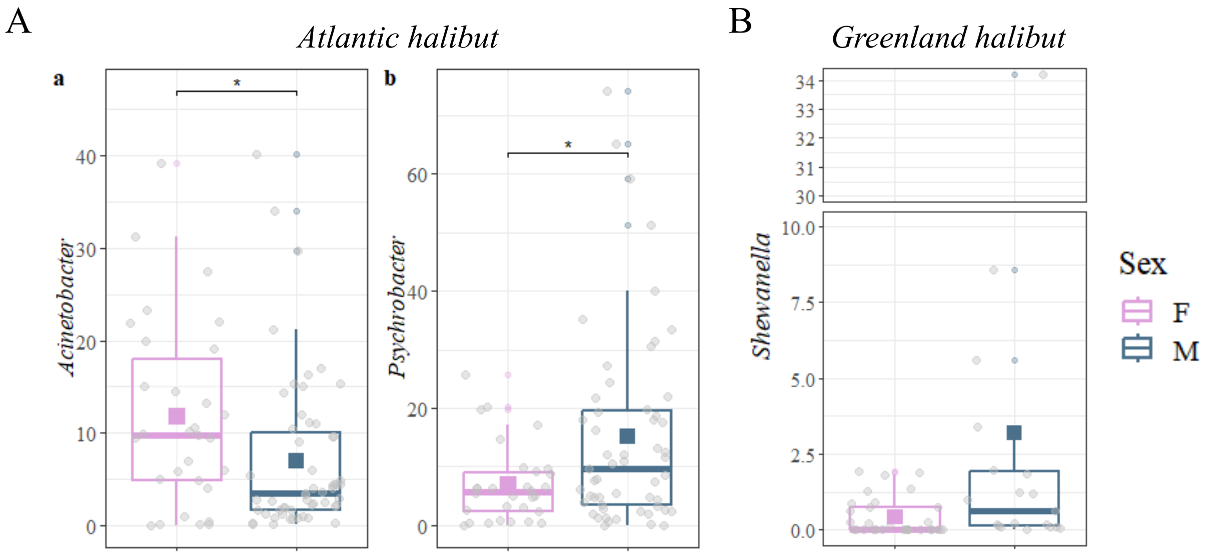

Figure S11: Relative abundance (%) of the discriminative taxa in the blood of male and female Atlantic (A) and Greenland (B) halibuts. Significant differences between sex were measured using the Wilcoxon-Mann-Whitney test. (*) p < 0.05; (**) p < 0.01; (***) p < 0.001.

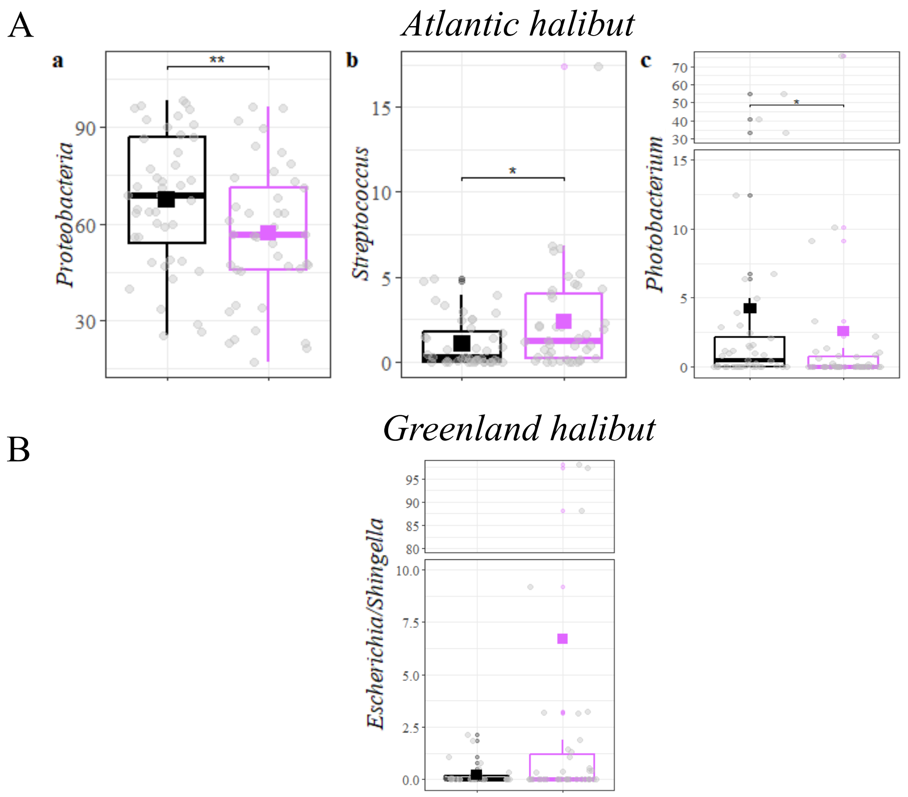

Figure S12: Relative abundance (%) of the discriminative taxa in the blood microbiome of according to condition classes. Discriminative taxa for (**A**) Atlantic and Greenland (**B**) halibut in bad (K>1) (black boxes) and good (K>1) (purple boxes) conditions. Significant differences between condition classes were tested with the Wilcoxon-Mann-Whitney test. (*) p < 0.05; (***) p < 0.001

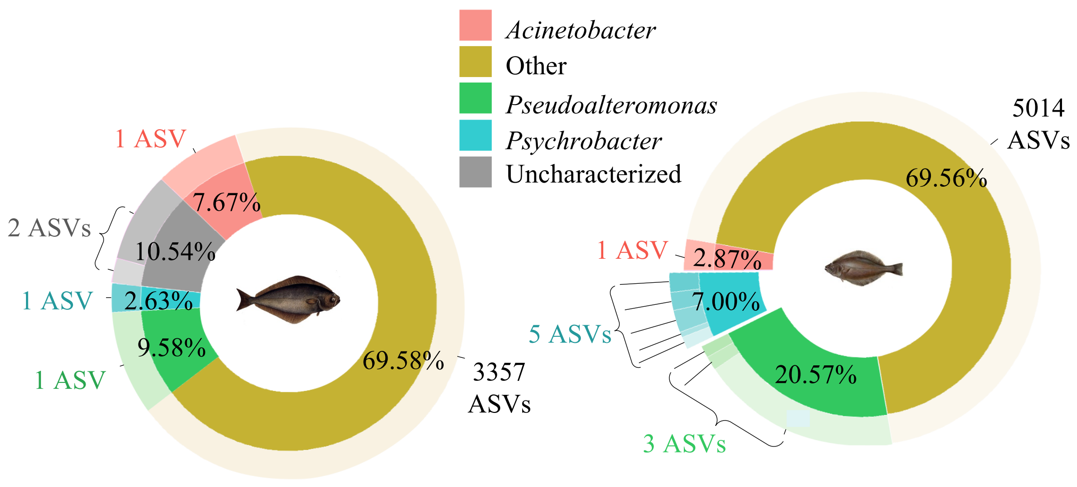

Figure S13: **Mean relative abundance of the core ASVs of the halibuts' circulating microbiome.** Mean relative abundance (%) of the core ASVs (70% prevalence) present the circulating microbiome of the Atlantic halibut (*H. hippoglossus*) and the Greenland halibut (*R. hippoglossoides*). The mean relative abundance is given in each pie. Atlantic halibut, n = 86, Greenland halibut, n = 97.

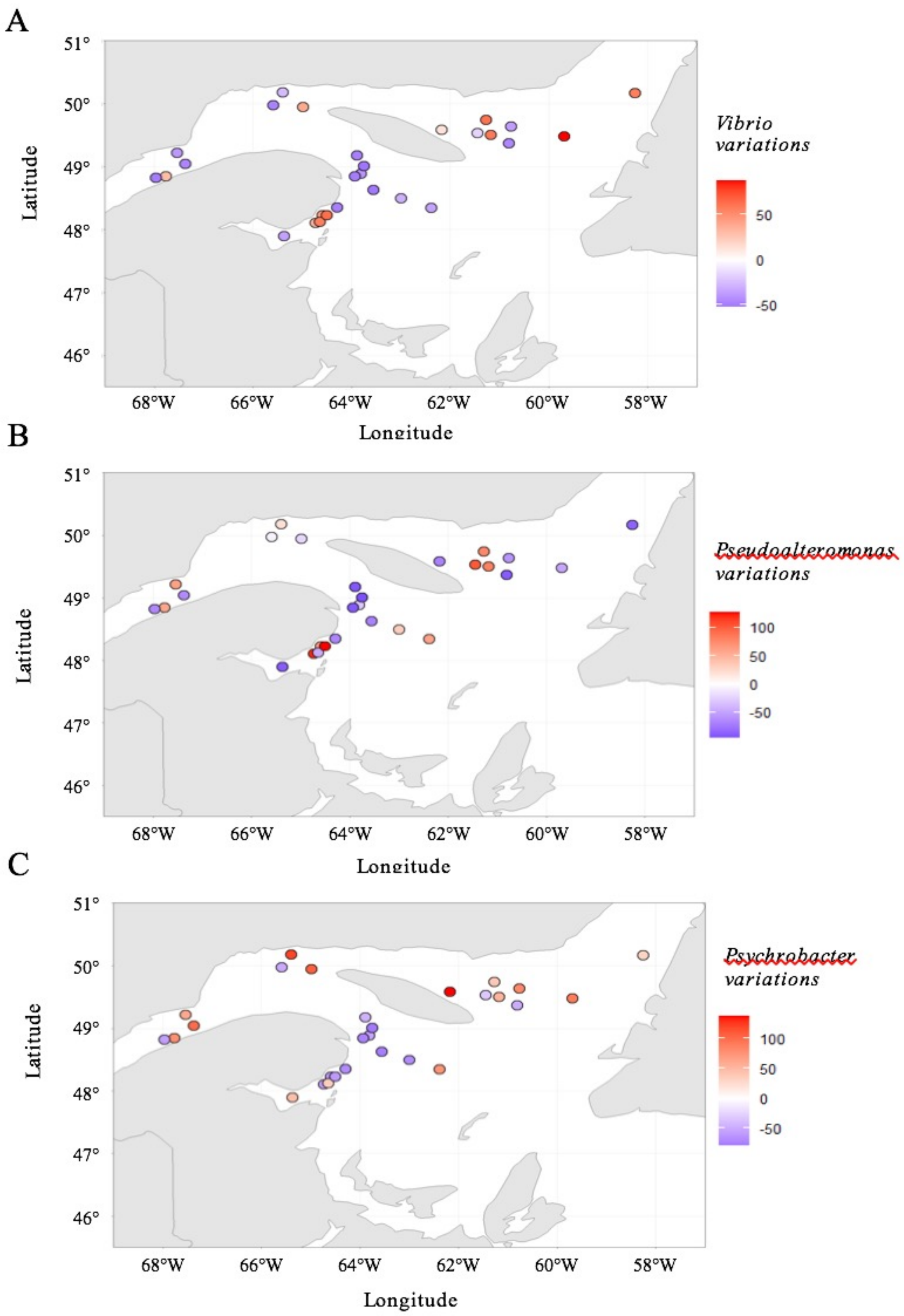

Figure S14: Maps of the variations around the mean flatted by a squared root in the Greenland halibut (*R. hippoglossoides*) blood microbiome core genera. A. *Vibrio* B. *Pseudoalteromonas*. C. *Psychrobacter.*

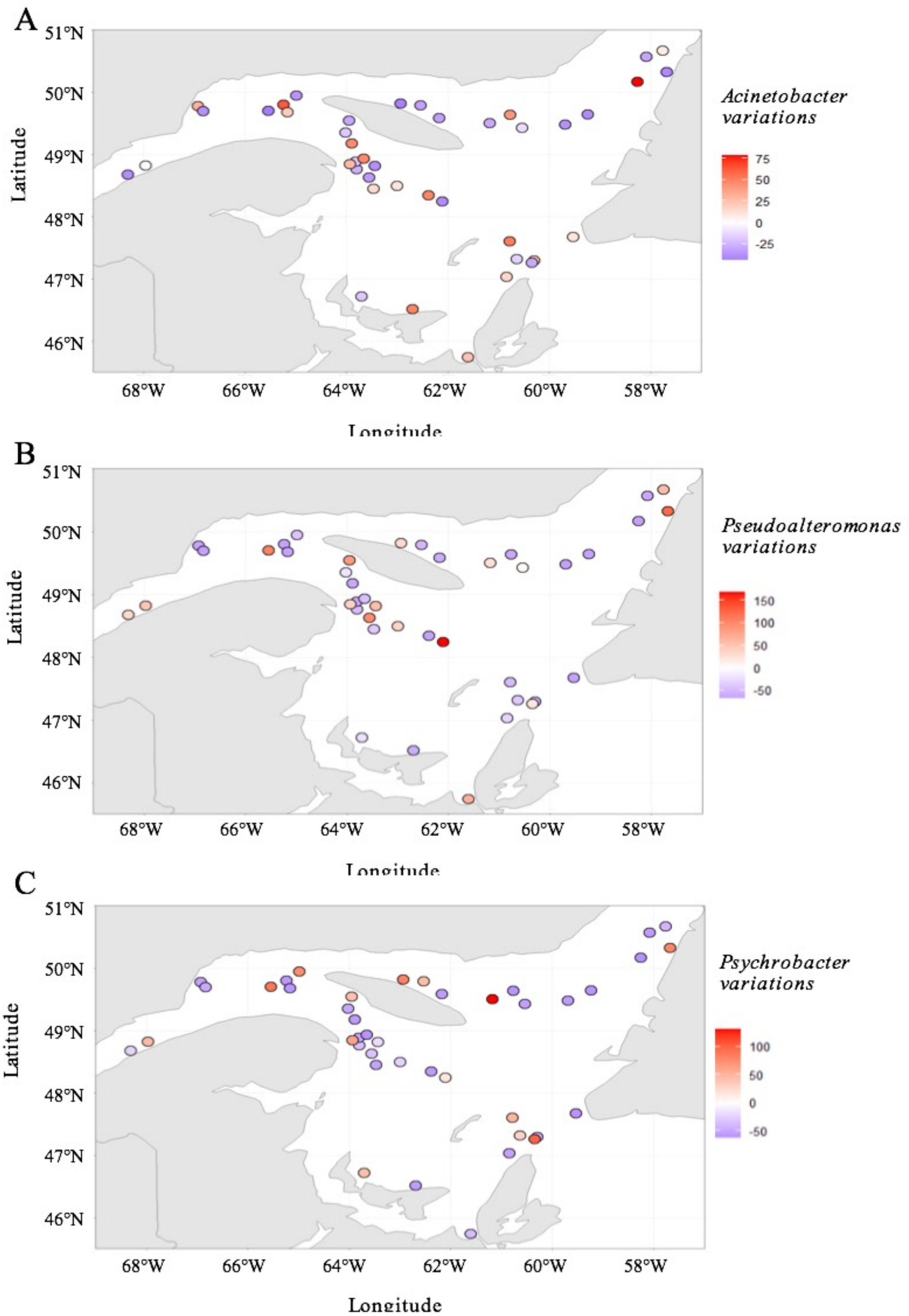

Figure S15: Maps of the variations around the mean flatted by a squared root in the Atlantic halibut (*H. hippoglossus*) blood microbiome core genera. A. *Acinetobacter* B. *Pseudoalteromonas*. C. *Psychrobacter*.
